# Multi-trophic markers illuminate the understanding of the functioning of a remote, low coral cover Marquesan coral reef food web

**DOI:** 10.1038/s41598-021-00348-w

**Published:** 2021-10-25

**Authors:** Pauline Fey, Valeriano Parravicini, Daniela Bănaru, Jan Dierking, René Galzin, Benoit Lebreton, Tarik Meziane, Nicholas V. C. Polunin, Mayalen Zubia, Yves Letourneur

**Affiliations:** 1 UMR ENTROPIE (UR-IRD-CNRS-IFREMER-UNC), LabEx « Corail », Université de La Nouvelle-Calédonie, BP R4, 98851 Nouméa Cedex, New Caledonia; 2 CRIOBE, PSL Research University, USR 3278 EPHE-CNRS-UPVD, LabEx « Corail », Université de Perpignan, Avenue Paul Alduy, 66860 Perpignan Cedex, France; 3 Mediterranean Institute of Oceanography, UM 110 (AMU-UTV-CNRS-IRD), Campus de Luminy, Case 901, 13288 Marseille Cedex 9, France; 4 GEOMAR Helmholtz Centre for Ocean Research, Research Division Marine Ecology, Düsternbrooker Weg 20, 24105 Kiel, Germany; 5 UMR LIENSs 7266 (CNRS-ULR), Institut du Littoral Et de L’environnement, 2 rue Olympe de Gouges, 17000 La Rochelle, France; 6 Laboratoire BOREA, Muséum National d’Histoire Naturelle, CNRS 7208, IRD 207-SU-UCN-UA, Muséum National d’Histoire Naturelle, 61 rue Buffon, 5 CP 53, 75231 Paris Cedex, France; 7 Newcastle University, School of Natural and Environmental Sciences, Newcastle-upon-Tyne, NE1 7RU UK; 8 UMR EIO (UPF-IRD-ILM-IFREMER), Université de La Polynésie Française, LabEx « Corail », BP 6570, 98702 Faa’a, Tahiti, French Polynesia

**Keywords:** Ecology, Ocean sciences

## Abstract

We studied the food web structure and functioning of a coral reef ecosystem in the Marquesas Islands, French Polynesia, characterized by low coral cover, high sea surface temperature and meso- to eutrophic waters. The Marquesas constitute a relevant ecosystem to understand the functioning of low diversity reefs that are also subject to global change. A multi-tracer assessment of organic matter pathways was run to delineate ecosystem functioning, using analysis of fatty acids, bulk and compound specific stable isotope analysis and stable isotopes mixing models. Macroalgae and phytoplankton were the two major food sources fueling this food web with, however, some marked seasonal variations. Specifically, zooplankton relied on phytoplankton-derived organic matter and herbivorous fishes on macroalgae-derived organic matter to a much higher extent in summer than in winter (~ 75% *vs.* ~ 15%, and ~ 70 to 75% *vs.* ~ 5 to 15%, respectively) . Despite remarkably high δ^15^N values for all trophic compartments, likely due to local dynamics in the nitrogen stock, trophic levels of consumers were similar to those of other coral reef ecosystems. These findings shed light on the functioning of low coral cover systems, which are expected to expand worldwide under global change.

## Introduction

Increasing anthropogenic and climatic stressors on Earth ecosystems have motivated a widespread interest in understanding the contribution of species to ecosystem functioning and energy flows between ecological compartments such as trophic guilds. Among marine environments, coral reefs are by far the most diverse ecosystems, hosting thousands of species forming vast, complex and very poorly resolved interaction networks that influence the structure, the functioning and the resilience of the ecosystems^1^. However, understanding the processes that drive ecosystem functioning and productivity requires determination of the major sources of organic matter fueling these systems^2^, and the stocks of nutrients^3^, and quantification of the numerous consumer compartments that rely on sources of organic matter^4, 5^. Elucidating the major energetic pathways that fuel ecosystems across the complexity of the interaction network is crucial not only to assess food web functioning and the resilience of coral reefs but also to anticipate unexpected cascading effects and undesirable ecological surprises related to global changes^6–8^.

Several studies have improved our understanding about flows of energy within coral reefs^4, 9–13^ but the processes involved, the species that support them and the ways these species interact are scarcely resolved. For instance, the high diversity of sources of organic matter available to primary consumers complicates the characterization of energy flows^2, 3, 14^. Carbon sources may have several origins, continental or marine, and can come from coastal, pelagic or deep waters, and also from living organisms or detrital matter^15, 16^. Oceanic phyto- and zoo-plankton may also be imported into reefs by currents, and then consumed by phyto- and zoo-planktivorous fish^17, 18^. While benthic sources represent an important carbon source to reef species^4, 12, 19^, numerous species rely principally or exclusively on planktonic sources^20^ and even non-planktivorous species may rely on oceanic production^12^. However, in several ecosystems, benthic production and/or terrestrial inputs can also be major contributors to the energy flow in food webs^2, 3, 21^.

The Marquesas Islands, one of the most isolated archipelagos on Earth, may provide important insights into the functioning of coral reefs characterized by low diversity and low coral cover. Decreasing coral cover and diversity are expected in many reef systems in the world facing increasing anthropogenic pressures^22–26^. Marquesas Islands coral reefs could not track sea level rise during the last post-glacial period^27^, and paleo-reefs can now be found at ~ 60–130 m depth, while modern shallow reefs are essentially rocky reefs with low coral cover. Other distinctive features of the Marquesas include the absence of *Acropora* spp. corals, which are common across other Polynesian coral reefs^28^. On one of the main islands of the Marquesas, Nuku Hiva, mean coral cover is only ~ 5%^29^. Other environmental conditions that make the Marquesas a highly pertinent system for the study of the functioning of their food web are their warm waters and high nutrient levels. The sea surface temperature is usually relatively high all year round (average 28–30°C^3, 28^), and sometimes higher during ENSO events, and thus substantially warmer than on several other reef systems like those located at higher latitudes and experiencing wider seasonal differences. Surface waters have high loads of nitrate (NO_3_^−^) and phosphate (PO_4_^−^), with concentrations higher than 1 μM and 0.3 μM, respectively, which exceed ~ 100-fold those measured in the South Pacific subtropical gyre^30^. This nutrient richness promotes the development of a high phytoplankton biomass around the Marquesan coasts over the year^30^.

In coral reef systems worldwide, the questions of ecosystem functioning, food web structure and function and organic matter sources fueling production are gaining attention^4, 11, 13, 31^. Here we characterize the major energetic pathways that fuel the unusual, low coral cover ecosystem of the Marquesas and assess the extent to which this pelagic production penetrates the coral reef food web. We analysed the trophic relationships among the food web components (sources of organic matter, primary consumers and higher trophic level consumers up to mesopredators) using multiple and complementary trophic markers. We combined information from carbon (δ^13^C) and nitrogen (δ^15^N) bulk stable isotope compositions, amino acid compound-specific isotopes compositions (δ^15^N) (AA CSIA) and fatty acids data. These three trophic have evidently yet to be combined to analyse any coral reef ecosystems, although they are capable of providing highly complementary information. Fatty acids are used to assess how the various sources of organic matter are integrated by primary consumers (herbivores) and then by secondary consumers, AA-CSIA provide information on both the assimilated baseline and the length of food chains, while bulk stable isotope analyses allow to describe the global structure of the food web. The combination of these methods allowed us to address five major questions: (i) What are the main sources of organic matter that fuel the food web of the Marquesas coral reefs, and (ii) how is this organic matter integrated by primary consumers? What is (iii) the trophic structure of this system, and (iv) the ‘baseline’ allowing us to assess the length of the food chain? (v) Is the uptake of the major sources of organic matter consistent across seasons? We then interpreted the observed patterns in the light of previous observations on other reef systems to draw inferences about possible coral reef functioning in future warming and possibly more eutrophic tropical oceans.

## Material and methods

### Study site and sampling

Field work was carried out in August 2016 (austral winter) and in March 2017 (austral summer) in southeastern Nuku Hiva (8°54’S, 140°02’W; Marquesas Islands, French Polynesia). The abiotic (rainfall, winds, temperature, hydrodynamics, etc.) and biotic (benthic and pelagic communities) conditions around the islands are well known^3, 28, 32^.

We sampled the major sources of organic matter (OM), primary consumers (invertebrates and fish) and several higher trophic-level consumers (invertebrates and fish), up to mesopredators. The sampling of OM sources, already described in detail^2^, comprise the particulate OM-hereafter POM mainly consisting of pico-nano phytoplankton–(n = 49), sedimentary OM-hereafter SOM-(n = 47), micro-phytoplankton-hereafter phytoplankton-(n = 49), 11 different locally abundant algae (algal turf and macroalgae, n = 73) and detrital terrestrial material-hereafter DTP-derived from tree leaves transported by rivers (n = 16).

The sampled consumers were 14 invertebrate species (sponges, ascidians, echinoderms, gastropods, bivalves and crustaceans-214 individuals in total), zooplankton (n = 49), and 29 fish species (n = 523). Invertebrates were collected by handpicking during scuba diving, in order to obtain 5–10 individuals per species and per season. For zooplankton, a 125 µm mesh-size WP2 net was used for a vertical tow in the water column (from ~ 40–50 m depth to the surface). Fish were collected by spearfishing or using an anesthetic (i.e. eugenol diluted at 10% in alcohol), in both seasons. All samples were identified to the lowest possible taxonomic level and were kept in ice chests during sampling and (except for POM, phyto- and zoo-plankton) immediately stored at − 20 °C until analysis.

First, we restricted our analysis to species representative of well-known trophic groups across the trophic-level gradient in order (i) to determine how the organic matter is assimilated by primary consumers, and (ii) to assess the length of the food chain. We selected nine primary consumers to study the integration of OM sources. Among these species, three were filter-feeders (the oyster *Pinctada margaritifera*, an unidentified ascidian, the sponge *Spheciospongia* sp.), one was a phytoplankton browser (zooplankton either 300–500 µm or 1000–2000 µm in size), and five were herbivore-detritivores (the gastropods *Mauritia* spp., the surgeonfishes *Acanthurus nigricans* and *Ctenochaetus marginatus*, and the parrotfishes *Scarus koputea* and *S. rubroviolaceus*). We also selected eight secondary-tertiary consumers expected to be at the top of the benthic food webs. These species were one gastropod, *Conus conco*, and seven fish: the snappers *Lutjanus bohar*, *L. gibbus*, and *L. kasmira*, the moray-eel *Enchelycore pardalis*, the scorpionfish *Scorpaenopis possi*, and the groupers *Cephalopholis argus* and *Epinephelus fasciatus*.

Then, we extended our analysis to all the species for which samples were available, i.e. the 14 invertebrate and 29 fish species collected. We defined the trophic position of all individuals in order to assess their position in the food web relative to the baseline, i.e. linked to OM sources.

Thus, the first analysis, focused on well-known trophic groups, allowed us to determine how the OM is incorporated and what the length of the food chain is. Then, the inclusion of all species allowed depiction of the food-web structure.

### Bulk stable isotope analyses

These analyses were run to get a general picture of the food web structure, the role of the OM sources and of various primary and secondary-tertiary consumers within the food web. The carbon and nitrogen stable isotopes (respectively δ^13^C and δ^15^N) were used in combination; δ^13^C give information on the origin(s) of the organic matter source(s) used by consumers^33^ and δ^15^N is a proxy of trophic level^34^, thus allowing a depiction of the food webs in bivariate isotopic space^35^.

A piece of the thallus was sampled for algae, soft muscle for all mollusks, and dorsal white muscle for fish^36^,  ~ 2–5 g in each case. For ascidians and sponges, ~ 5–10 g pieces, excluding external theca for ascidians, were taken from each individual. For zooplankton, several entire individuals were grouped to obtain the 5 mg dry mass required for analysis.

Carbon and nitrogen bulk stable isotope compositions (δ^13^C and δ^15^N) were determined in all samples. Sediment was dried and reduced to a fine powder using a mortar and pestle. POM was collected on precombusted GF/F filters (porosity 0.7 µm), and dried. Plant (algae, DTP) and animal (zooplankton, invertebrates and fish) samples were freeze-dried and ground to fine powder. Approximately 1 mg of powder was precisely weighed and encapsulated for plant/animal samples, and 15–20 mg for SOM and 15–30 mg for POM and phytoplankton (matter extracted by scrapping the filter). For POM and SOM, two subsamples were analyzed: one was acidified to eliminate inorganic carbonates from the sample and used for δ^13^C analysis^37^, while the other was not acidified and used for δ^15^N analysis^43^. The other samples were analyzed without prior treatment. Samples were analyzed through continuous-flow isotope-ratio mass spectrometry with a Flash 2000 elemental analyzer equipped with the Smart EA option (Thermo Scientific, Milan, Italy), coupled with a Delta V Advantage isotope ratio mass spectrometer with a Conflo IV interface (Thermo Scientific, Bremen, Germany) at the Littoral, Environment and Societies Joint Research Unit stable isotope facility (LIENSs) at the University of La Rochelle (France). Isotope compositions were expressed in the δ notation as parts per mil (‰) as deviations from an international standard (i.e. Vienna Pee Dee Belemnite for carbon and atmospheric N_2_ for nitrogen) following the formula:

$$\delta {\mathrm{X}} = \left[ {\left( {{\mathrm{R}}_{{\mathrm{sample}}} /{\mathrm{R}}_{{\mathrm{standard}}} } \right) - 1} \right]{ \times }1000$$

where X is ^13^C or ^15^N, R is the corresponding ratio (^13^C/^12^C or ^15^N/^14^N). Calibration was done using reference materials (USGS-24, -61, -62, IAEA-CH6, -600 for carbon; USGS-61, -62, IAEA-N2, –NO-3, -600 for nitrogen). The analytical precision of the measurements was 0.1‰ for carbon and < 0.15‰ for nitrogen based on analyses of USGS-61 and USGS-62 used as laboratory internal standards.

### Compound-specific amino acid stable isotope analyses

These analyses were run to (i) help define the ‘baseline’ of the food web through the use of sources amino acids, and (ii) to assess food chain length through both trophic amino acids and bulk stable isotopes. Sr-AA δ ^15^N values capture the real baseline isotopic composition much better than those of bulk δ^15^N because the latter value pool several amino acids, whereas Sr-AA δ^15^N values do not fluctuate over different trophic levels, thus capturing the baseline isotope composition of the food web without biases^5^.

Compound-specific δ^15^N values of amino acids (AA-CSIA) were derived from eight selected species having high bulk δ^15^N values (see above). This included the gastropod and the seven secondary-tertiary consumer fish species (total 44 samples for both seasons). Samples were prepared by acid hydrolysis followed by derivatization to produce trifluoroacetic amino acid esters (TFAA) using standard methods^38^. The δ^15^N values of the TFAA derivatives of amino acids were analyzed using a isotope ratio mass spectrometer (Delta V Plus, Thermo Scientific, Bremen, Germany) interfaced with a gas chromatograph (Trace GC 1300, Thermo Scientific, Bremen, Germany) through a GC IsoLink combustion furnace, and liquid nitrogen cold trap at the University of Davis (California, USA). Measured isotopic values were corrected relative to known δ^15^N values of norleucine, the internal reference material. All samples were analyzed in triplicate. δ^15^N values of the glycine and phenylalanine were then measured for source amino acids (δ^15^N_Sr-AA_) in order to assess the baseline δ^15^N values (i.e. the δ^15^N of the primary producers at the base of the food web^5, 39^). So, knowing characteristics of main organic matter sources by one hand (i.e. the ‘baseline’), and knowing species at the top of the food web on the other hand (i.e. the eight selected species), we expected to be able to assess the food chain length.

### Trophic positions

We estimated the trophic position (hereafter TP) of several individuals using a combination of AA-CSIA and bulk SIA. In particular, we used the δ^15^N values from bulk SIA of target individuals and from AA-CSIA to characterize the baseline. Then, TP was calculated as follows^40^:

1 $${\text{TP}}_{{\text{x}}} = \frac{{{\updelta }^{15} {\text{N}}_{{\text{x}}} - {{ \delta }}^{15} {\text{N}}_{{{\text{baseline}}}} }}{{{\text{TEF}}}} + {\text{ TP}}_{{{\text{baseline}}}}$$

where x is the species of interest and TEF is the trophic enrichment factor between trophic levels. In our case, we set the TEF at 3.4 ‰ as commonly done in the marine coastal environment^40^. Because the source amino acids (glycine, phenylalanine) represent the δ^15^N value of the baseline, a TP_baseline_ of 1 was used.

### Fatty acid analyses

These analyses were run to assess the integration of OM sources by primary producers and possibly the use of these herbivores by secondary-tertiary consumers.

Fatty acid (FA) were analysed in the OM sources (SOM, river and marine POM, DTP, algae and phytoplankton^2^) and in nine primary consumers: zooplankton (n = 23), four invertebrate species (n = 54) and four fish species (n = 56). Lipids were extracted^41^, using 5–20 mg of material for POM, 20–30 mg for DTP, algae, phyto- and zooplankton, sponges and ascidians, 30–40 mg for mollusks and fish and 1 g for SOM. Tricosanoic acid (23:0), was added to each sample as an internal standard to measure the FA concentrations. Fatty acid methyl esters (FAMEs) were quantified by gas chromatography (Varian 3800-GC), using a flame ionization detector. FAs were identified by comparing retention times with those of a commercial standard (Supelco) and confirmed using a mass spectrometer coupled to a gas chromatograph (Varian 450-GC; Varian 220-MS). Fatty acid concentrations are expressed as % of total FAs, or as absolute concentrations in mg.g^−1^.

Functional groups of fatty acids, classified by degree of unsaturation, were used as indicators of different types of organic matter^42, 43^: the saturated fatty acids (SFAs; e.g. 14:0, 16:0 and 18:0), monounsaturated fatty acids (MUFAs; e.g. 16:1ω7 and 18:1ω9), polyunsaturated fatty acids (PUFAs; e.g. 20:4ω6, 20:5ω3 and 22:6ω3) and branched fatty acids (BrFAs; e.g. iso-15:0 and anteiso-15:0). Among SFAs, the long-chain SFAs (C24-C28) are widely used as an indicator of detrital terrestrial plant (DTP) material^44^. Bacteria and cyanobacteria are also rich in SFAs^45^. C16 and C18 MUFAs are found in algae^46^, bacteria^47^ and zooplankton^48^. PUFAs with an omega-3 (ω3) or omega-6 (ω6) terminal end are named essential fatty acids (EFAs) because animals are considered typically to acquire them solely from diet^45^. PUFAs are abundant in fresh plankton material (major components of phytoplankton), but these FAs are rapidly degraded in the water column and the sediment^49, 50^. Iso- and anteiso-FAs (BrFAs) are solely synthesized by bacteria and therefore are deemed to be a good indicator of these microorganisms^47, 51^.

### Assessment of OM integration by primary consumers

Assessment of food source uses by primary consumers was achieved through two complementary methods. The first approach is based on the close relationship between the isotope compositions of a consumer and its food sources^52^. The contributions of the different sources to the diets of primary consumers (i.e. macroalgae, algal turf, phytoplankton, POM and/or SOM) were determined using a Bayesian mixing model and the SIAR package^53^. OM sources with similar isotope composition were pooled to avoid incorrect determination of their relative contribution. Models were run for 200,000 iterations, the burn-in was set at 50,000 iterations and a one-fifteenth thinning was applied. This model provides a range of solutions regarding the proportions of the different sources (i.e. macroalgae, algal turf, phytoplankton, POM and/or SOM). To reduce potential bias associated with the definition of a single trophic enrichment factor (i.e. 3.4‰), we also used TEF (Δ^13^C and Δ^15^N) values adapted to the feeding strategies and trophic positions of the studied taxa (Suppl. Figure S1). The determination of Δ^13^C and Δ^15^N values was based on the isotope compositions of the primary consumers (see results) in combination with previous findings^3^ on OM sources. The herbivore-detritivores showed TEFs values of 1.60 ± 1.87‰ for Δ^13^C and 4.28 ± 1.05‰ for Δ^15^N, while filter-feeders and zooplankton had 1.24 ± 1.16‰ for Δ^13^C and 2.71 ± 0.85‰ for Δ^15^N.

The second approach was based on the exploration of the links between FAs and primary consumers through a principal component analysis (PCA), using the 25 individual FAs that had an average proportion greater than 1% within at least one group of samples. The statistics and graphical representations were performed using R version 3.4.4^54^, using ggplot2, gridExtra, ggrepel, vegan, FactoMiner and car packages. Identification of FAs bio-indicating particular OM sources allowed us to assess the importance of the integration of various potential OM sources by primary consumers.

### Ethical statement

This research received no specific grant from any commercial or not-profit sectors. No coral habitat was degraded during this research. Sample collection was permitted by the French Polynesian government (authorization number: 681/MCE/ENV), which also approved the experimental protocols. All methods were carried out in accordance with relevant guidelines and regulations. All methods were performed in compliance with the ARRIVE guidelines and regulations for ethical treatment of animals^55^.

## Results

### OM sources in the Marquesan coastal ecosystem

As the data on the different potential sources of organic matter were already widely presented and discussed in a previous article^42^, only a brief outline is given hereafter. The mean δ^13^C values ranged from − 23.9 ± 1.7‰ for algal turf to − 16.4 ± 2.0‰ for macroalgae (^3^, Suppl. Table 1). The mean δ^15^N values ranged from 11.6 ± 0.9‰ for macroalgae to 15.0 ± 1.8‰ for phytoplankton (^3^, Suppl. Table S1).

### Primary consumers: trophic marker characteristics and use of OM sources

Mean δ^13^C values ranged from − 19.6 ± 2.7 (*Acanthurus nigricans*) to − 14.0 ± 1.3‰ (*Ctenochaetus marginatus*) in winter, and from − 20.2 ± 0.5 (small zooplankton) to − 13.9 ± 1.2‰ (*C. marginatus*) in summer (Table 1). Mean δ^15^N values ranged from 12.6 ± 0.9 (small zooplankton) to 19.1 ± 1.0‰ (*C. marginatus*) in winter, and from 14.2 ± 0.4 (*Pinctada margaritifera*) to 17.4 ± 1.4‰ (*C. marginatus*) in summer. The parrotfish and cypraeid species did not differ significantly from each other in mean δ^13^C and δ^15^N values (Table 1, Kruskal–Wallis tests, *P* > 0.05) and were hereafter pooled with *Mauritia* spp. and Scarinae, respectively.

**Table 1:** Bulk δ^13^C and δ^15^N values (means ± standard deviation) of nine selected primary consumers.

		Code	n	δ^13^C‰	δ^15^N‰
**Invertebrates**					
Ascidiidae	Ascidian	Asc	2	− 17.8 ± 1.2	14.6 ± 0.3
			***15***	− ***18.7*** ± *** 0.4***	***15.1*** ± ***1.1***
Clionaidae	*Spheciospongia* sp.	Spsp	28	− 18.1 ± 0.5	15.8 ± 0.6
			***16***	− ***17.7*** ± ***0.3***	***16.1*** ± ***0.5***
Pteriidae	*Pinctada margaritifera*	Pima	7	− 16.7 ± 0.4	15.2 ± 0.5
			***17***	− ***17.0*** ± ***0.3***	***14.5*** ± ***0.4***
Cypraeidae	*Mauritia* spp.	Maspp	5	− 15.3 ± 1.1	15.8 ± 0.2
			***8***	− ***16.4*** ± ***0.6***	***16.6*** ± *** 0.9***
Zooplankton	300–500 µm	Zoo-300	9	− 19.6 ± 0.3	12.6 ± 0.9
			***12***	− ***20.2*** ± ***0.5***	***14.9*** ± ***1.1***
	1000–2000 µm	Zoo-1000	12	− 19.4 ± 0.5	14.1 ± 0.5
			***16***	− ***19.2*** ± ***0.7***	***17.4*** ± ***0.5***
**Fish**					
Acanthuridae	*Acanthurus nigricans*	Acni	11	− 19.6 ± 2.7	17.0 ± 0.8
			***14***	− ***17.7*** ± ***1.9***	***16.1*** ± ***0.9***
	*Ctenochaetus marginatus*	Ctma	25	− 14.0 ± 1.3	19.1 ± 1.0
			***13***	− ***13.9*** ± ***1.2***	***17.4*** ± ***1.5***
Scarinae	*Scarus koputea*	Scar	4	− 17.7 ± 0.2	19.0 ± 0.3
			***9***	− ***14.9*** ± ***1.2***	***16.4*** ± ***1.2***
	*S. rubroviolaceus*		8	− 16.6 ± 1.2	18.8 ± 1.1

Summer with bold italic, winter without bold italic.

We detected 57 FAs, of which 35 had concentrations greater than 1% (see Suppl. Table S2 for details). The two size groups of zooplankton had similar fatty acid compositions (Kruskal–Wallis test, *P* > 0.05) and were thus pooled hereafter. The sponge *Spheciospongia* sp. represented an outlier with extremely high percentages of several SFAs, MUFAs and mostly PUFAs (Suppl. Table S2), and was therefore removed from further analyses.

**Figure 1:**
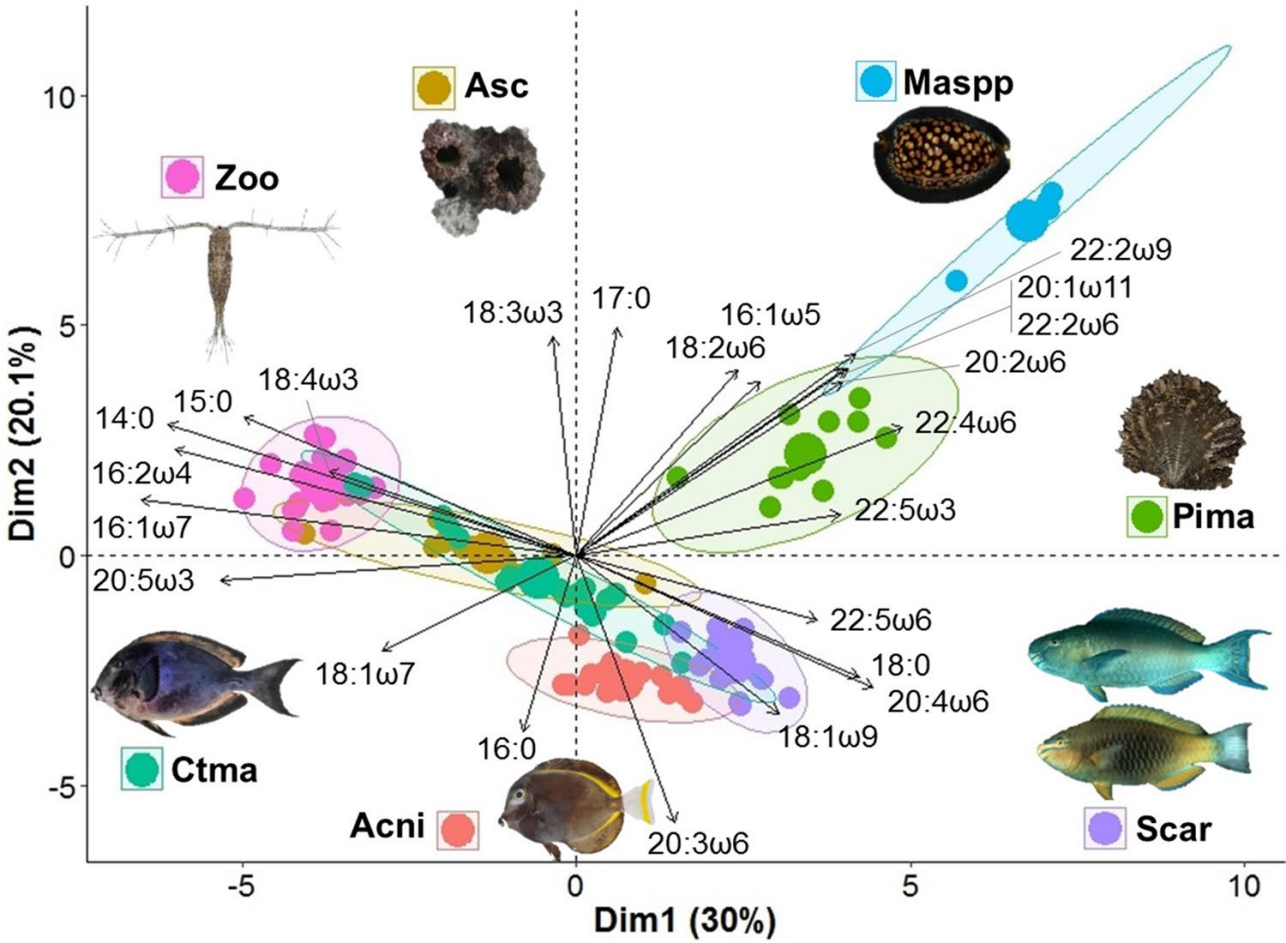
PCA of fatty acid (FA) compositions of the invertebrates (excluding sponges) and fishes. Codes of species: Asc: ascidians, Pima: *Pinctada margaritifera*, Maspp: *Mauritia* spp., Zoo: zooplankton, Acni: *Acanthurus nigricans*, Ctma: *Ctenochaetus marginatus*, Scar: *Scarus koputea* and *S. rubroviolaceus*. Only FAs with a mean percentage > 1% in at least one species were considered (see text).

Different primary consumers were characterized by different FA profiles (Fig. 1, Suppl. Table S2). For instance, zooplankton was mostly characterized by some SFAs (14:0 and 15:0), MUFAs (16:1ω7) and PUFAs (16:2ω4 and 20:5ω3) (Fig. 1). Scarinae were mostly characterized by 16:0 and 18:0 (SFA), 18:1ω9, 20:4ω6, 22:5ω6 (PUFAs), and 22:6ω3, whereas *Acanthurus nigricans* was characterized by 16:0 (SFA) and 18:1ω9 (Fig. 1, Suppl. Table S2).

**Figure 2:**
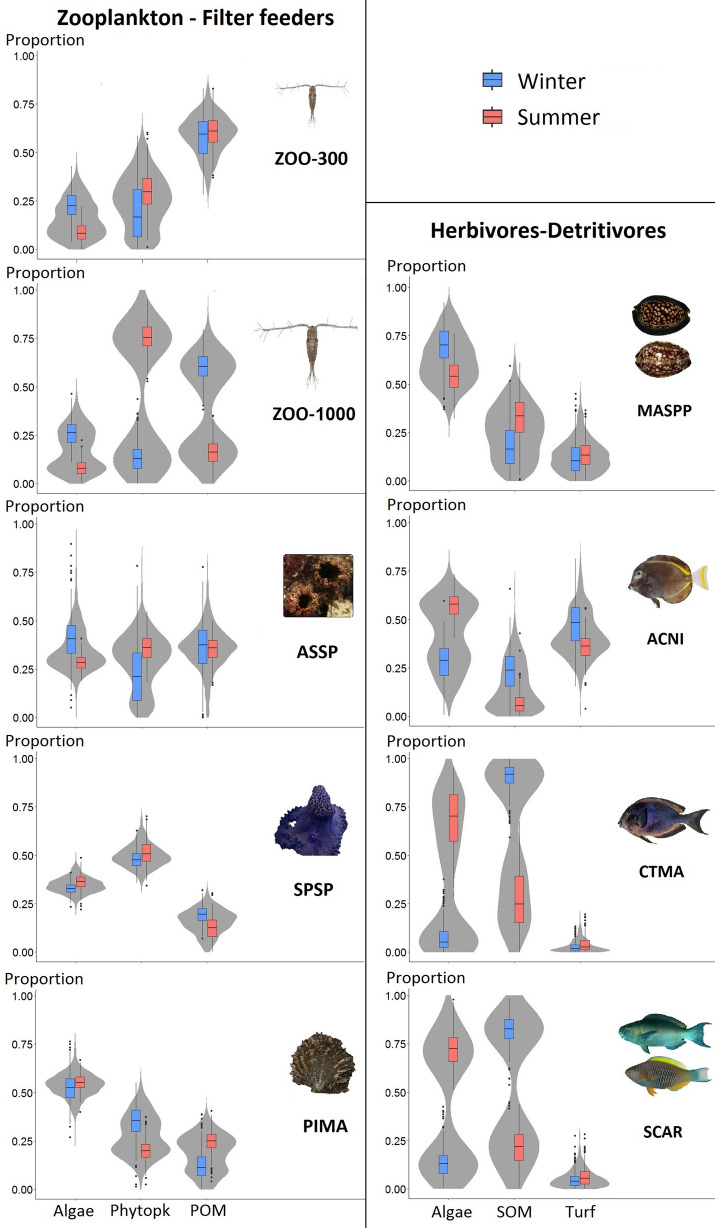
Relative proportions of the different OM sources for filter-feeders and zooplankton (left panel) and for herbivore-detritivores (right panel), based on SIAR mixing model outputs. Phytopk: Phytoplankton; POM: particulate organic matter; SOM: sedimentary organic matter. Violin plots represent the posterior distribution shape of the data (density estimation) in grey; boxplots represent median, interquartile ranges (red and blue boxes, depending on season), 95% credibility intervals (thin line) and outliers (black dots). ZOO-300: zooplankton 300–500 μm, ZOO-1000: zooplankton 1000–2000 μm, ASSP: ascidians, SPSP: *Spheciospongia* sp., PIMA: *Pinctada margaritifera,* ACNI: *Acanthurus nigricans*, CTMA: *Ctenochaetus marginatus*, SCAR: Scarinae (*Scarus koputea* and *S. rubroviolaceus*), MASPP: *Mauritia* spp.

We detected differences in the use of major sources of OM among seasons for zooplankton and herbivore-detritivores (Fig. 2). Small zooplankton mostly relied on POM in both seasons (~ 60% on average, credibility interval (CI) ~ 30–85%), with phytoplankton contributing ~ 20–30% to isotope composition (CI ~ 0–60%). Large zooplankton relied mostly on POM during winter (~ 60%, CI ~ 40–85%) and on phytoplankton during summer (~ 75%, CI ~ 50–100%) (Fig. 2). Similar seasonal differences occurred in the filter-feeders, although with a much lower amplitude. The major sources of OM for ascidians were phytoplankton, algae and POM in relatively equal proportions (~ 25–40% depending on seasons); the sponge *Spheciospongia* sp. did not use a lot of POM, and *Pinctada margaritifera* relied mainly on macroalgae, and less on POM and phytoplankton (Fig. 2). SOM and macroalgae were important OM sources for herbivore-detritivores but with a strong seasonal contrast in some cases, ranging from ~ 10% (*Acanthurus nigricans* in summer) to ~ 90% (*Ctenochaetus marginatus* in winter) and from ~ 10% (*C. marginatus* in winter) to ~ 75% (*C. marginatus* in summer), respectively (Fig. 2). Algal turf was a marginal OM source for Scarinae and *C. marginatus*, contributing ~ 10–15% to the isotope composition of *Mauritia* spp. and up to ~ 50% (in winter) to that of *A. nigricans*.

### Secondary consumers trophic markers and the general structure of the food web

**Figure 3:**
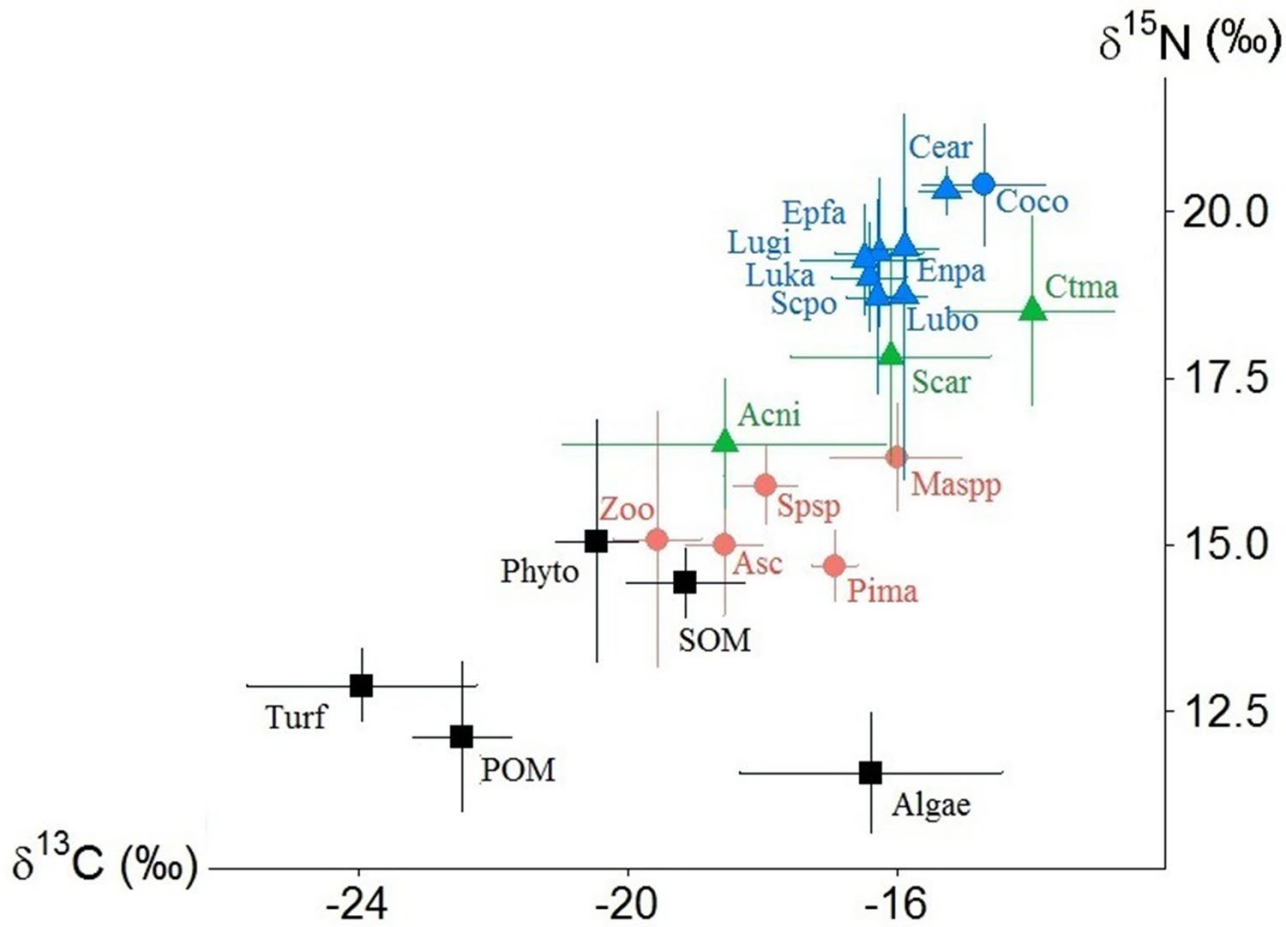
Plot of δ^15^N against δ^13^C values (means ± standard deviations) of sources of organic matter (black squares, see Fey et al. 2020 for details), nine primary consumers (red circles: invertebrates, green triangles: fish), and eight secondary consumers (blue circle: invertebrate, blue triangles: fish), both seasons pooled. Turf: algal turf, Phyto: phytoplankton, Algae: macroalgae, POM: particulate organic matter, SOM: sedimentary organic matter, Asc: ascidians, Spsp: *Speciospongia* sp., Pima: *Pinctada margaritifera*, Maspp: *Mauritia* spp., Zoo: zooplankton, Acni: *Acanthurus nigricans*, Ctma: *Ctenochaetus marginatus,* Scar: Scarinae, Coco: *Conus conco*, Lubo: *Lutjanus bohar*, Lugi: *L. gibbus*, Luka: *L. kasmira*, Enpa: *Enchelycore pardalis*, Scpo: *Scorpaenopsis possi*, Cear: *Cephalopholis argus*, Epfa: *Epinephelus fasciatus.*

Based on bulk isotope compositions of OM sources, nine primary consumers and eight secondary-tertiary consumers, we were able to delineate the general structure of the major energetic pathways of the Marquesan coastal food web (Fig. 3). For secondary-tertiary consumers, mean bulk δ^13^C values ranged from − 16.5 to − 14.7‰, and those of δ^15^N values ranged from to 18.7 to 20.4‰ (Fig. 3). The highest mean δ^15^N values were measured in the endemic gastropod *Conus conco* and the grouper *Cephalopholis argus* (20.4 ± 0.9‰ and 20.3 ± 0.4‰, respectively). For most secondary-tertiary consumers, differences between seasons were often low and non-significant (Suppl. Table S3). Other sampled consumer species not included in Fig. 3 for clarity fitted well within this network based on their δ^13^C and δ^15^N values (Suppl. Table S3).

On average, the mean δ^15^N value of Sr-AA (phenylalanine and glycine) was 11.6 ± 3.4‰ with values ranging from 7.1‰ for glycine in *Lutjanus bohar* to 17.0‰ for phenylalanine in *Conus conco* (Table 2). Differences in Sr-AA δ^15^N values among species were more evident for glycine than for phenylalanine.

**Table 2:** Source amino acids (AA) δ^15^N values (‰) of eight secondary consumers (means ± standard deviation), both seasons pooled.

Species	Code	n	Phenylalanine	Glycine	Mean
*Conus conco*	Coco	6	15.2 ± 3.9	17.0 ± 1.6	16.1 ± 3.0
*Lutjanus gibbus*	Lugi	6	13.0 ± 1.7	7.9 ± 1.6	10.5 ± 3.1
*Lutjanus kasmira*	Luka	6	12.3 ± 3.3	8.8 ± 1.9	10.5 ± 3.2
*Lutjanus bohar*	Lubo	4	12.0 ± 5.2	7.1 ± 4.2	9.5 ± 5.1
*Enchelycore pardalis*	Enpa	4	11.9 ± 1.9	13.2 ± 0.7	12.4 ± 1.7
*Scorpaenopsis possi*	Scpo	6	12.2 ± 3.0	10.5 ± 1.8	11.4 ± 2.5
*Cephalopholis argus*	Cear	6	13.6 ± 1.9	8.2 ± 1.2	10.9 ± 3.2
*Epinephelus fasciatus*	Epfa	6	11.9 ± 1.9	10.9 ± 2.2	11.4 ± 2.0
Total		44	12.8 ± 3.0	10.5 ± 3.6	11.6 ± 3.4

**Figure 4:**
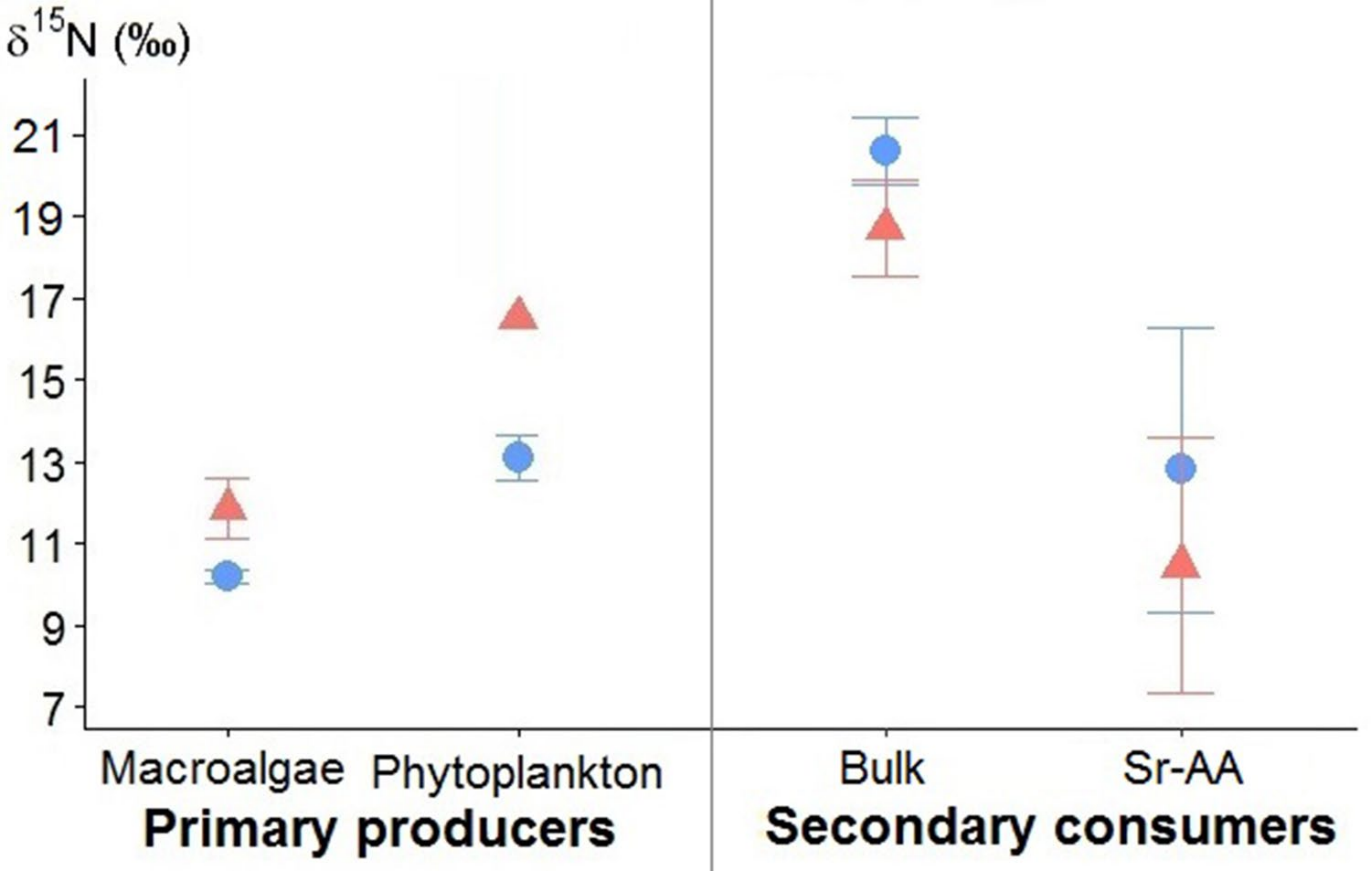
Seasonal variations of bulk δ^15^N values (means ± standard deviation) for the most integrated primary producers (left panel), and bulk, and Sr-AA (phenylalanine and glycine) for our eight targeted secondary consumers, all species pooled (right panel). Blue dots: winter, red triangles: summer.

The eight secondary consumers also displayed seasonal differences in both their mean bulk δ^15^N and Sr-AA δ^15^N values (Fig. 4), although not statistically significant, mean values were higher in winter than summer, in contrast to what was observed for bulk δ^15^N values of phytoplankton and macroalgae (Fig. 4).

### Baseline and trophic positions

Although the isotope compositions of primary consumers evidenced the use of several OM sources (Fig. 2), the relatively high concentrations of the fatty acid 20:4ω6, a biomarker of macroalgae, in all primary consumers (even for zooplankton although at lower concentrations) allowed us to identify in the macroalgae the main baseline to estimate TP. The Sr-AA δ^15^N_phe-gly_ values obtained for the eight mesopredators were also used.

**Figure 5:**
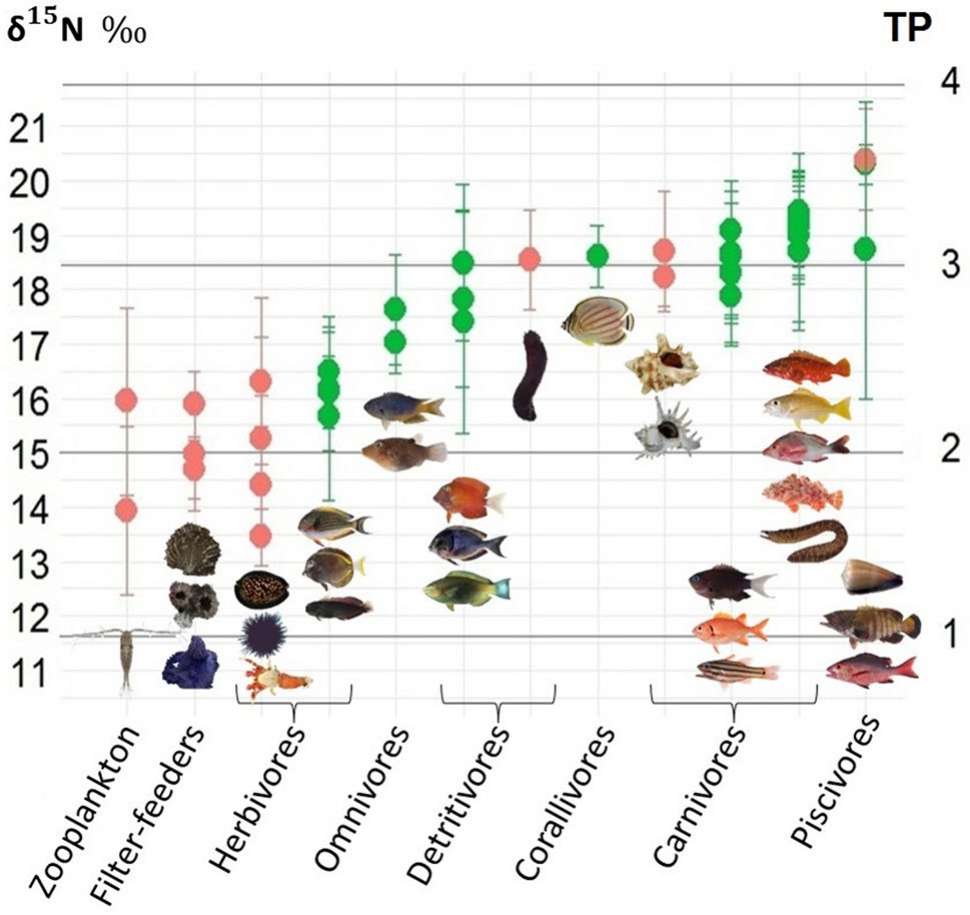
Relationships between mean bulk δ^15^N values and trophic positions (TPs) calculated with Sr-AA δ^15^N_phe-gly_ values and with macroalgae as the main source of organic matter. Each dot corresponds to the mean TP for a species. Black circles for sources of organic matter, red circles for invertebrates, and green circles for fish.

The mean bulk δ^15^N values of all individuals (n = 797) and species (n = 43) belonging to different trophic groups (Suppl. Table S3) provided a detailed picture of their trophic positions in the food web (Fig. 5). Most primary consumers (i.e. filter-feeders, zooplankton and herbivores) displayed a TP of ~ 2–2.3, omnivores and detritivores had a TP of ~ 2.4–3, carnivores were at a TP ~ 3–3.2 and piscivores showed a TP of ~ 3.2–3.6, with the highest TP being in *Conus conco* and *Cephalopholis argus* (3.57 ± 0.27 and 3.55 ± 0.23, respectively; Fig. 5). This global picture was consistent across seasons for all trophic groups, despite higher δ^15^N values in summer than in winter (Suppl. Figure S2).

## Discussion

We provide the first combined application of bulk and compound specific stable isotope as well as fatty acid data to a coral reef food web, and elucidate food web functioning including major energetic pathways, OM sources and trophic positions of multiple species on a Marquesan coral reef. Despite the peculiar ecological characteristics of these coral reefs (warm water, high nutrient levels, low coral cover as well as reduced biodiversity compared to many other reefs), fatty acid, C and N stable isotope data evidenced that this system maintains a high productivity fueled by phytoplankton and zooplankton, most likely of pelagic origin. Moreover, despite unusually high δ^15^N values for all groups from OM sources up to mesopredators, trophic positions and food chain lengths were comparable to those documented for other coral reef ecosystems^56, 57^. Since conditions experienced in the Marquesas today are projected for many other reef systems in the future, this study offers valuable insights into the future of changing coral reefs.

The outputs from mixing models highlighted that macroalgae, phytoplankton, POM and SOM are the main sources of OM for the consumers of the Marquesas, while turf algae had a comparatively minor role. Algal turf is often described as a major OM sources in coral reefs, especially for herbivorous fish^4, 10, 58, 59^. However, at Nuku Hiva, it contributed only ~ 15% to herbivores, although in *Acanthurus nigricans* its contribution to biomass reached ~ 50% in winter. In a complex coral reef system in New Caledonia, four major sources of OM used by consumers were evidenced^2^, i.e. algal turf (the most important one), sedimentary OM mixed with macroalgae, particulate OM, and -to a lower extent- detritus and seagrass. In a Caribbean coral reef, algal turf is also the main OM source for consumers^57^. The reasons for a marginal importance of algal turf in Marquesas Islands remain unclear. Algal turf may lack important nutritive elements^60^ inducing herbivores to shift their diet to other items, such as macroalgae. In contrast to macroalgae, algal turf in Marquesas displayed a high C/N ratio (~ 18^3^), much higher than the value of ~ 12 usually considered as characteristic of refractory organic matter^61^. This suggests a low nutritional value of this food item and might explain why algal turf was little used in Marquesas, despite its wide distribution. The reason for such a C/N ratio remains unclear and requires further investigations.

**Figure 6:**
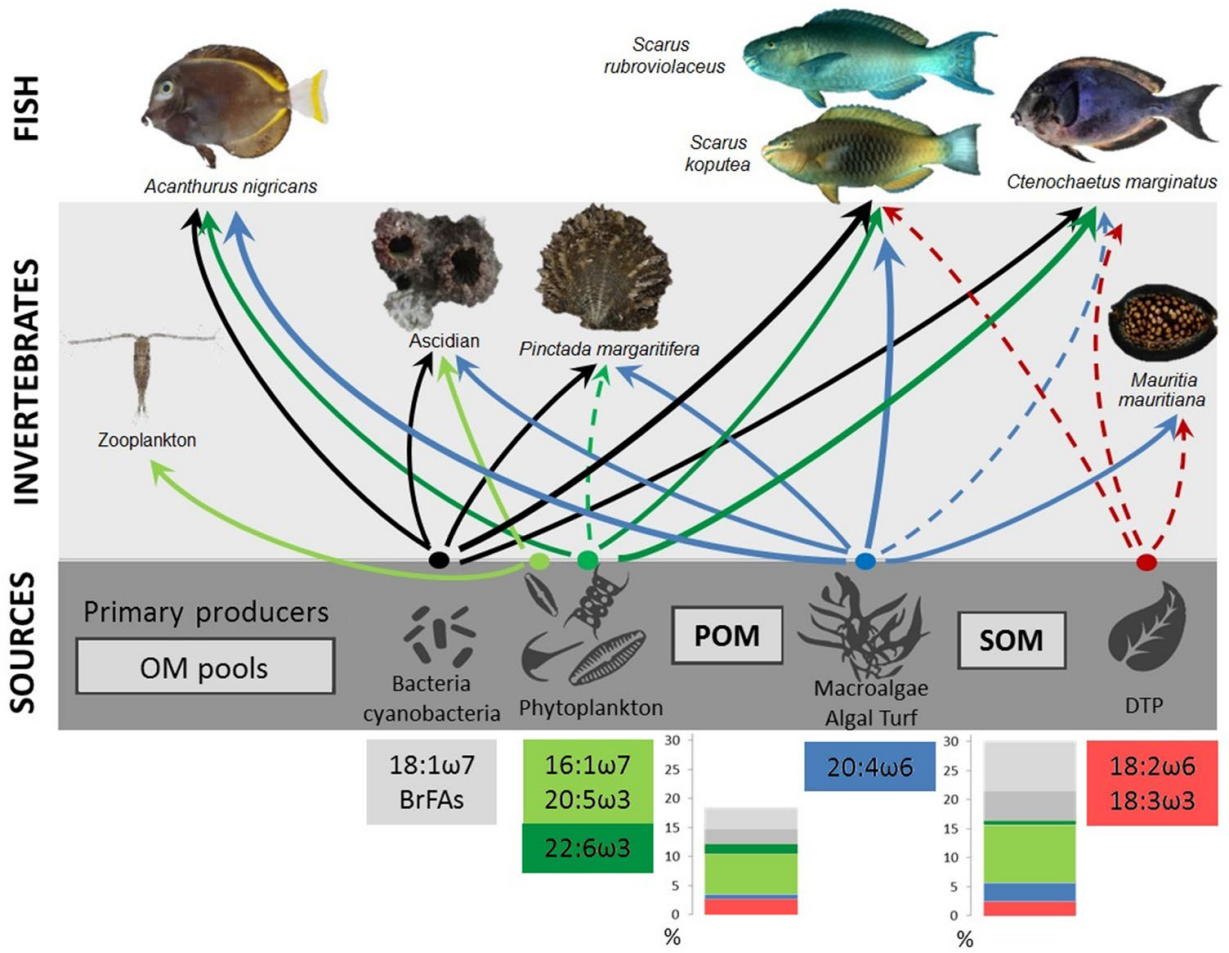
Schematic view of the integration of main OM sources by primary consumers, through fatty acid trophic markers. The relative importance of such integration is indicated by arrows (from dotted lines for minor role, to thick lines for important role). POM: particulate organic matter, SOM: sedimentary organic matter, DTP: detrital terrestrial plant material. For OM pools (POM and SOM), the % indicate the relative importance of primary producers (bacteria, phytoplankton, DTP etc.) assessed by major FAs trophic markers.

The FA compositions of the primary consumers also highlighted the importance of the macroalgae in the food web. Indeed, we detected a high abundance of the FAs 18:3ω3 and 20:4ω6 in all primary consumers (Fig. 6). Diatom markers (20:5ω3, 16:1ω7 and 16:2ω4) were also present in primary consumers, especially zooplankton, ascidians, and the detritivore-herbivores *Acanthurus nigricans* and *Ctenochaetus marginatus*. Although we did not sample bacteria/cyanobacteria, markers typical of these organisms (FAs 15:0, 17:0, 17:0iso, and 18:1ω7) were found in ascidians, *Pinctada margaritifera* and all fish. This suggest a potential role of bacteria in the Marquesan food web. Bacteria have not been previously shown to contribute substantially to the OM that support reef fishes. Therefore, future studies are needed to clarify their contribution. Cyanobacteria have been recorded on several herbivores fish diets such as in *Scarus* spp.^62^. Our FA data however cannot distinguish between filamentous turf-forming species and non-colonial small species. The strong contribution of the fatty acid 22:6ω3, a marker of dinoflagellates found in phytoplankton^3^, to the total FAs of zooplankton, *P. margaritifera*, *C. marginatus* and *Scarus* spp. indicated that phytoplankton was an important food source not only for zooplankton, but also for filter-feeders and some herbivores. However, we did not identify an important contribution of phytoplankton-derived OM for Acanthuridae and Scarinae, even though these taxa occasionally feed on zooplankton and indirectly assimilate phytoplankton-derived OM^63^. We suggest that the link between this OM source and these primary consumers is probably indirect, through deposition and accumulation of recently dead phytoplankton (or zooplankton) on sediments and macroalgae.

The compound specific stable isotopes (Sr-AA δ^15^N_phe-gly_) allowed us to define the δ^15^N values of the food web baseline. These values were higher in winter than in summer (Fig. 4), in contrast to the seasonal variability recorded in bulk isotope compositions. This apparent contradiction may be due to a temporal lag. Indeed, the isotope compositions of the source amino acids were measured on consumer, which likely have a longer turn-over than primary producers. Also, the estimated δ^15^N values of the food web baseline obtained with bulk stable isotope data reflected relatively recent variations of isotope composition (i.e. isotope composition of the OM sources at the time of collection, typical of the sampled season). In contrast, the amino acid isotope compositions provided a value of the sources referred to a longer time frame corresponding to the renewal time of the tissues analyzed, i.e. ~ 3 months before sampling for fish muscle.

**Figure 7:**
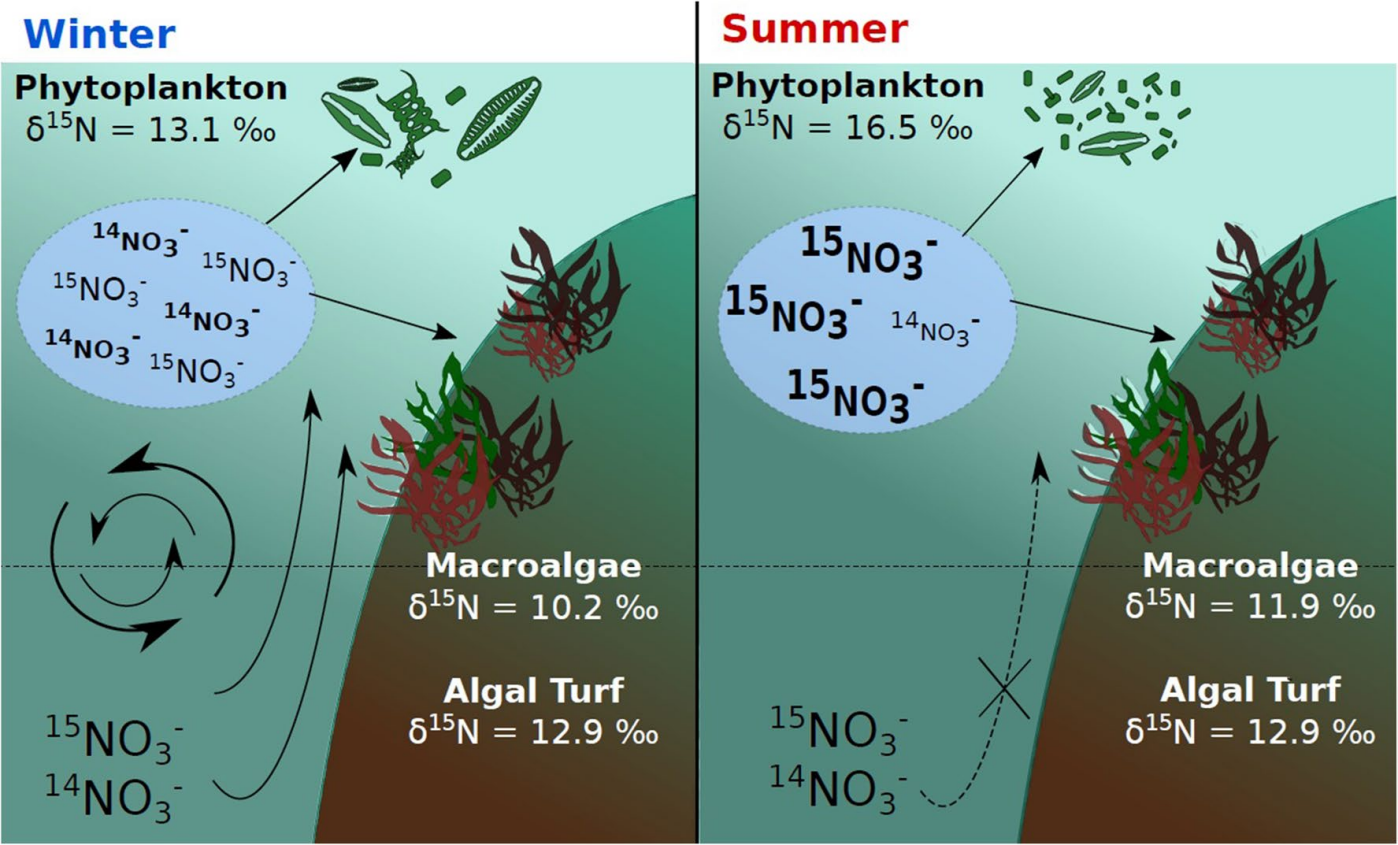
Schematic view of seasonal processes that could explain seasonal differences in nitrogen isotope compositions in primary producers.

The unusually high bulk δ^15^N values of OM sources up to mesopredators suggest a high δ^15^N value of the food web baseline that then propagates through the system. It is thus essential to understand why this pattern exists and why δ^15^N values remain high across seasons despite winter / summer fluctuations (Fig. 7). The δ^15^N values measured in the primary producers in the Marquesas were ~ 8–10‰ higher than in other Pacific sites^2, 9, 10, 64^. These δ^15^N values are probably due to the ^15^N enrichment of nutrient reservoirs in Marquesan waters, and seasonal changes in the strength of hydrodynamic processes such as eddies and upwelling^32^. δ^15^N values increase in declining nitrate reservoirs, due to the more rapid assimilation of nitrates containing the light isotope (^14^N) during photosynthesis^65^. The high phytoplankton biomass throughout the year in the Marquesas Islands^30^, and its use of nitrate (NO_3_^−^) helps explain the ^15^N enrichment of the reservoirs of residual nutrients^66^. In the Marquesas, the high nutrient intake in summer may promote significant fractioning and enhance the δ^15^N values of the residual nitrate^65, 67^ (Fig. 7). By assimilating ^15^N-enriched nitrates, primary producers such as phytoplankton and macroalgae thus increase their δ^15^N values. This is not the case for turf algae that sustain similar δ^15^N values over seasons. Other processes may drive the ^15^ N enrichment of nitrates and primary producers, such as higher bacterial denitrification in summer^65^, a possibly important role of bacteria in the functioning of the Marquesan coral reef ecosystem. Mineralization, nitrogen fixation, assimilation, nitrification and/or denitrification processes influence the ^15^N composition of both inorganic (e.g. N_2_, NO_3_^−^, NO_2_^−^) and organic nitrogen species^50^.

The assessment of trophic positions (TPs) of organisms within food webs is essential to understand ecosystem structure and functioning^68^, although a thorough quantification remains challenging given the large number of factors involved (e.g. ontogeny of species, change of dietary regime). The δ^15^N values of the source amino acids phenylalanine and glycine indicate that the Marquesan coastal food web is mostly supported by macroalgae. However, the high variability around the mean implies that this finding should be considered with some caution. Seasonal fluctuations in phytoplankton isotope composition in the Marquesas make this compartment an important food source for several primary consumers^3^. This suggests that coupled pelagic-benthic processes likely drive the general characteristics and properties of OM sources over seasons. The TP values found in other studies are close to ours, despite very different bulk δ^15^N values. For instance in a Caribbean coral reef, the five carnivorous fish having the highest TPs reached values of ~ 3.3, for bulk δ^15^N values of ~ 9.5‰^57^. In a Polynesian coral reef, those values reached ~ 3.5 and ~ 11‰, respectively^56^. Overall, the TPs we obtained for the different species or trophic groups are very similar to those from these studies, implying similar food chain lengths.

The overall increase in the TP for all consumers in summer did not alter this general pattern. Under the hypothesis that the Marquesas may be considered a present-day reference for degraded reefs, the Nuku Hiva data suggest that the length of the food chain may remain unchanged in future coral reefs subject to anthropogenic degradations, as also observed in Mexico^57^. However, systems with similar food chain lengths may strongly differ in energy flows. For instance, the seasonality in TP values in the Marquesas, which were mostly apparent for omnivores and some carnivores (Suppl. Figure S2), also reflected a change in the use of OM and in the energy pathway within food webs. Coral losses alter isotope compositions of coral reef fish^69^. Since environmental changes may produce substantial effects on fish abundance and biomass^70^, ongoing coral reefs degradations may drastically affect fluxes of energy and ecosystem functioning^7^. While some coral reefs appeared to be resilient to disturbances, mostly thanks to herbivory^71^, a shift from coral towards algal reefs seems likely in the coming decades^72–74^. On such reefs, the role of macroalgae and plankton driven by nutrient enrichments especially for nitrogen might grow. Although the present Nuku Hiva data were mainly qualitative, a greater potential role of bacterial / cyanobacterial communities as significant sources of OM in future coastal systems needs more consideration than it has had to date^75, 76^.

## Conclusions

Although some characteristics appear to be similar to other tropical reef systems, in many respects the food web functioning of the Marquesas Islands coral reef ecosystems is quite atypical. Fatty acid data highlighted that the roles of several sources of OM, such as bacteria / cyanobacteria which are generally difficult to collect in classical food web studies, may have been underestimated in other reef systems. Pelagic-derived OM may exceed the key-role of macroalgae as the main baseline of this food web. Similar conclusions regarding the importance of pelagic-derived organic matter as a food source for coral reefs were drawn from other studies^13, 77^. This reinforces the hypothesis of a food web based on pelagic-benthic coupling^3^, and suggests that such functioning might become an increasingly important characteristic in future coral reefs. Despite the unusually high δ^15^N values in all compartments of the studied food web, the trophic positions of consumers were comparable to other reef systems. Although this suggests that food chain lengths might not be much affected by the expected loss of corals, seasonal and more long-term variations in the use of OM by consumers will likely affect energy flows. Much further research is needed to better assess how coral reef flows of energy and organic matter might change in the next decades. The current Marquesa’ Islands coral reefs offer one plausible future scenario for functioning of this ecosystem. The resilience of the Marquesan reef ecosystem may be low and this raises concerns about its capacity to resist future changes.

## Supplementary Information

Supplementary Information 1.

Supplementary Information 2.

## References

[1] Folke C (2004). Regime shifts, resilience, and biodiversity in ecosystem management. Annu. Rev. Ecol. Evol. Syst..

[2] Briand MJ, Bonnet X, Goiran C, Guillou G, Letourneur Y (2015). Major sources of organic matter in a complex coral reef lagoon: Identification from isotopic signatures (δ^13^C and δ^15^N). PLoS ONE.

[3] Fey P (2020). Sources of organic matter in an atypical phytoplankton rich coral ecosystem, Marquesas Islands: composition and properties. Mar. Biol..

[4] Briand MJ, Bonnet X, Guillou G, Letourneur Y (2016). Complex food webs in highly diversified coral reefs: insights from δ^13^C and δ^15^N stable isotopes. Food Webs.

[5] Bierwagen SL, Heupel MR, Chin A, Simpfendorfer CA (2018). Trophodynamics as a tool for understanding coral reef ecosystems. Front. Mar. Sci..

[6] Halpern BS (2008). A Global map of human impact on marine ecosystems. Science.

[7] Hughes TP (2018). Global warming transforms coral reef assemblages. Nature.

[8] Hughes TP (2019). Global warming impairs stock–recruitment dynamics of corals. Nature.

[9] Wyatt ASJ, Waite AM, Humphries S (2012). Stable isotope analysis reveals community-level variation in fish trophodynamics across a fringing coral reef. Coral Reefs.

[10] Letourneur Y (2013). Identifying carbon sources and trophic position of coral reef fishes using diet and stable isotope (δ^15^N and δ^13^C) analyses in two contrasted bays in Moorea, French Polynesia. Coral Reefs.

[11] Zhu Y, Newman SP, Reid WDK, Polunin NVC (2019). Fish stable isotope community structure of a Bahamian coral reef. Mar. Biol..

[12] McMahon KW, Thorrold SR, Houghton LA, Berumen ML (2015). Tracing carbon flow through coral reef food webs using a compound-specific stable isotope approach. Oecologia.

[13] Skinner C (2021). Offshore pelagic subsidies dominate carbon inputs to coral reef predators. Sci. Adv..

[14] Mann KH (1988). Production and use of detritus in various freshwater, estuarine and coastal marine ecosystems. Limnol. Oceanogr..

[15] Antonio B, Maria Teresa A-O, Manuel V (2006). Phytoplankton and macrophyte contributions to littoral food webs in the Galician upwelling estimated from stable isotopes. Mar. Ecol. Prog. Ser..

[16] Gazeau F, Smith SV, Gentili B, Frankignoulle M, Gattuso J-P (2004). The European coastal zone: characterization and first assessment of ecosystem metabolism. Est. Coast. Shelf Sci..

[17] Hamner WM, Jones MS, Carleton JH, Hauri IR, Williams DM (1988). Zooplankton, planktivorous fish, and water currents on a windward reef face: great Barrier Reef, Australia. Bull. Mar. Sci..

[18] Hamner WM, Colin PL, Hamner PP (2007). Export-import dynamics of zooplankton on a coral reef in Palau. Mar. Ecol. Prog. Ser..

[19] Carassou L, Kulbicki M, Nicola TJR, Polunin NVC (2008). Assessment of fish trophic status and relationships by stable isotope data in the coral reef lagoon of New Caledonia, southwest Pacific. Aquat. Living Resour..

[20] Frédérich B, Fabri G, Lepoint G, Vandewalle P, Parmentier E (2009). Trophic niches of thirteen damselfishes (Pomacentridae) at the Grand Récif of Toliara, Madagascar. Ichthyol. Res..

[21] Riera P, Richard P (1996). Isotopic determination of food sources of *Crassostrea gigas* along a trophic gradient in the estuarine bay of Marennes-Oléron. Estuar. Coast. Shelf Sci..

[22] Hoegh-Guldberg O (1999). Climate change, coral bleaching and the future of the world's coral reefs. Mar. Fresh. Wat. Res..

[23] Bellwood DR, Hughes TP, Folke C, Nyström M (2004). Confronting the coral reef crisis. Nature.

[24] Bruno JF, Selig ER (2007). Regional decline of coral cover in the Indo-Pacific: Timing, extent, and subregional comparisons. PLoS ONE.

[25] Roff G (2014). Porites and the Phoenix effect: unprecedented recovery after a mass coral bleaching event at Rangiroa Atoll, French Polynesia. Mar. Biol..

[26] Hoey A (2016). Recent advances in understanding the effects of climate change on coral reefs. Diversity.

[27] Cabioch G (2008). Successive reef depositional events along the Marquesas foreslopes (French Polynesia) since 26 ka. Mar. Geol..

[28] Galzin, R., Duron, S. D. & Meyer, J. Y. *Biodiversité terrestre et marine des îles Marquises, Polynésie française*. (Société française d’Ichtyologie, 2016).

[29] SO CORAIL. *Site d'observation CORAIL*, https://sextant.ifremer.fr/record/le51de1b-7979-4487-b5d5-329394d166da (2018).

[30] Martinez, E., M., R. & Maamaatuaiahutapu, K. in *Biodiversité terrestre et marine des îles Marquises, Polynésie française* (eds Galzin R., Duron S.-D., & Meyer J.-Y) 123–136 (Société Française d’Ichtyologie, 2016).

[31] Houk P, Musburger C (2013). Trophic interactions and ecological stability across coral reefs in the Marshall Islands. Mar. Ecol. Prog. Ser..

[32] Raapoto H, Martinez E, Petrenko A, Doglioli AM, Maes C (2018). Modeling the Wake of the Marquesas Archipelago. J. Geophys. Res. Oceans.

[33] Vander Zanden MJ, Rasmussen JB (2001). Variation in δ^15^N and δ^13^C trophic fractionation: Implications for aquatic food web studies. Limnol. Oceanogr..

[34] De Niro MJ, Epstein S (1978). Influence of diet on the distribution of carbon isotopes in animals. Geochim. Cosmochim. Acta.

[35] Layman CA (2012). Applying stable isotopes to examine food-web structure: an overview of analytical tools. Biol. Rev..

[36] Pinnegar J, Polunin NVC (1999). Differential fractionation of d13C and d15N among fish tissues: implications for the study of trophic interactions. Funct. Ecol..

[37] De Niro MJ, Epstein S (1978). Influence of diet on the distribution of carbon isotopes in animals. Geochim. Cosmochim. Acta.

[38] Hannides CCS, Popp BN, Landry MR, Graham BS (2009). Quantification of zooplankton trophic position in the North Pacific Subtropical Gyre using stable nitrogen isotopes. Limnol. Oceanogr..

[39] Hannides CCS, Popp BN, Choy CA, Drazen JC (2013). Midwater zooplankton and suspended particle dynamics in the North Pacific Subtropical Gyre: a stable isotope perspective. Limnol. Oceanogr..

[40] Post DM (2002). Using stable isotopes to estimate trophic position: models, methods, and assumptions. Ecology.

[41] Meziane T (2007). Inter-specific and geographical variations in the fatty acid composition of mangrove leaves: implications for using fatty acids as a taxonomic tool and tracers of organic matter. Mar. Biol..

[42] Parrish, C. C. *et al.* in *Marine Chemistry* (ed P. J. Wangersky) 193–223 (Springer Berlin Heidelberg, 2000).

[43] Alfaro AC, Thomas F, Sergent L, Duxbury M (2006). Identification of trophic interactions within an estuarine food web (northern New Zealand) using fatty acid biomarkers and stable isotopes. Est. Coast. Shelf Sci..

[44] Meyers PA (1997). Organic geochemical proxies of paleoceanographic, paleolimnologic, and paleoclimatic processes. Org. Geochem..

[45] Dalsgaard, J., St. John, M., Kattner, G., Müller-Navarra, D. & Hagen, W. in *Advances in Marine Biology* Vol. 46 225–340 (Academic Press, 2003). 10.1016/s0065-2881(03)46005-7 14601414

[46] Volkman JK, Jeffrey SW, Nichols PD, Rogers GI, Garland CD (1989). Fatty acid and lipid composition of 10 species of microalgae used in mariculture. J. Exp. Mar. Biol. Ecol..

[47] Volkman JK, Johns RB, Gillan FT, Perry GJ, Bavor HJ (1980). Microbial lipids of an intertidal sediment—I. Fatty acids and hydrocarbons. Geochimica et Cosmochimica Acta.

[48] Lee RF, Hirota J, Barnett AM (1971). Distribution and importance of wax esters in marine copepods and other zooplankton. Deep Sea Res. A.

[49] Wakeham SG, Hedges JI, Lee C, Peterson ML, Hernes PJ (1997). Compositions and transport of lipid biomarkers through the water column and surficial sediments of the equatorial Pacific Ocean. Deep Sea Res. Part II.

[50] Budge SM, Parrish CC (1998). Lipid biogeochemistry of plankton, settling matter and sediments in Trinity Bay, Newfoundland. II. Fatty acids. Organic Geochem..

[51] Meziane T, Agata DF, Lee SY (2006). Fate of mangrove organic matter along a subtropical estuary: small-scale exportation and contribution to the food of crab communities. Mar. Ecol. Prog. Ser..

[52] Phillips DL, Gregg JW (2003). Source partitioning using stable isotopes: coping with too many sources. Oecologia.

[53] Parnell AC, Inger R, Bearhop S, Jackson AL (2010). Source partitioning using stable isotopes: Coping with too much variation. PLoS ONE.

[54] R Core Team. (R Foundation for Statistical Computing, Vienna, Austria, 2018).

[55] du Percie S, N.  (2020). Reporting animal research: explanation and elaboration for the ARRIVE guidelines 2.0. PLOS Biol..

[56] Page HM (2013). Stable isotopes reveal trophic relationships and diet of consumers in temperate kelp forest and coral reef ecosystems. Oceanography.

[57] Morillo-Velarde PS (2018). Habitat degradation alters trophic pathways but not food chain length on shallow Caribbean coral reefs. Sci. Rep..

[58] Bellwood DR, Choat JH (1990). A functional analysis of grazing in parrotfishes (family Scaridae): The ecological implications. Environ. Biol. Fish..

[59] Choat JH, Clements KD, Robbins WD (2002). The trophic status of herbivorous fishes on coral reefs. I: Dietary analyses. Mar. Biol..

[60] Dromard CR (2013). Resource use of two damselfishes, Stegastes planifrons and Stegastes adustus, on Guadeloupean reefs (Lesser Antilles): Inference from stomach content and stable isotope analysis. J. Exp. Mar. Biol. Ecol..

[61] Hedges JI (1986). Compositions and fluxes of particulate organic material in the Amazon River1. Limnol. Oceanogr..

[62] Nicholson GM, Clements KD (2020). Resolving resource partitioning in parrotfishes (Scarini) using microhistology of feeding substrata. Coral Reefs.

[63] Clements KD, German DP, Piché J, Tribollet A, Choat JH (2016). Integrating ecological roles and trophic diversification on coral reefs: multiple lines of evidence identify parrotfishes as microphages. Biol. J. Lin. Soc..

[64] Bradley CJ, Longenecker K, Pyle RL, Popp BN (2016). Compound-specific isotopic analysis of amino acids reveals dietary changes in mesophotic coral-reef fish. Mar. Ecol. Prog. Ser..

[65] Raimbault P, Garcia N, Cerutti F (2008). Distribution of inorganic and organic nutrients in the South Pacific Ocean-evidence for long-term accumulation of organic matter in nitrogen-depleted waters. Biogeosciences.

[66] Savoye N (2003). Dynamics of particulate organic matter d15N and d13C during spring phytoplankton blooms in a macrotidal ecosystem (Bay of Seine, France). Mar. Ecol. Prog. Ser..

[67] Montoya JP, McCarthy JJ (1995). Isotopic fractionation during nitrate uptake by phytoplankton grown in continuous culture. J. Plankton Res..

[68] Hussey NE (2014). Rescaling the trophic structure of marine food webs. Ecol. Lett..

[69] Letourneur Y, Briand MJ, Graham NAJ (2017). Coral reef degradation alters the isotopic niche of reef fishes. Mar. Biol..

[70] Graham NAJ (2011). Extinction vulnerability of coral reef fishes. Ecol. Lett..

[71] Viviani J (2019). Synchrony patterns reveal different degrees of trophic guild vulnerability after disturbances in a coral reef fish community. Divers. Distrib..

[72] Diaz-Pulido G, Gouezo M, Tilbrook B, Dove S, Anthony KRN (2011). High CO2 enhances the competitive strength of seaweeds over corals. Ecol. Lett..

[73] Koch M, Bowes G, Ross C, Zhang X-H (2013). Climate change and ocean acidification effects on seagrasses and marine macroalgae. Glob. Change Biol..

[74] Ainsworth TD (2016). Climate change disables coral bleaching protection on the Great Barrier Reef. Science.

[75] Jackson JBC (2001). What is natural in the coastal oceans?. Proc. Natl. Acad. Sci. USA.

[76] Bourne DG, Morrow KM, Webster NS (2016). Insights into the coral microbiome: underpinning the health and resilience of Reef ecosystems. Annu. Rev. Microbiol..

[77] Morais RA, Bellwood DR (2019). Pelagic subsidies underpin fish productivity on a degraded coral reef. Curr. Biol..

