# Author Correction: Multi-trophic markers illuminate the understanding of the functioning of a remote, low coral cover Marquesan coral reef food web

**DOI:** 10.1038/s41598-021-02477-8

**Published:** 2021-11-23

**Authors:** Pauline Fey, Valeriano Parravicini, Daniela Bănaru, Jan Dierking, René Galzin, Benoit Lebreton, Tarik Meziane, Nicholas V. C. Polunin, Mayalen Zubia, Yves Letourneur

**Affiliations:** 1 UMR ENTROPIE (UR‑IRD‑CNRS‑IFREMER‑UNC), LabEx « Corail », Université de La Nouvelle-Calédonie, BP R4, 98851 Nouméa Cedex, New Caledonia; 2 CRIOBE, PSL Research University, USR 3278 EPHE-CNRS-UPVD, LabEx « Corail », Université de Perpignan, Avenue Paul Alduy, 66860 Perpignan Cedex, France; 3 Mediterranean Institute of Oceanography, UM 110 (AMU-UTV-CNRS-IRD), Campus de Luminy, Case 901, 13288 Marseille Cedex 9, France; 4 GEOMAR Helmholtz Centre for Ocean Research, Research Division Marine Ecology, Düsternbrooker Weg 20, 24105 Kiel, Germany; 5 UMR LIENSs 7266 (CNRS-ULR), Institut du Littoral Et de L’environnement, 2 rue Olympe de Gouges, 17000 La Rochelle, France; 6 Laboratoire BOREA, Muséum National d’Histoire Naturelle, CNRS 7208, IRD 207-SU-UCN-UA, Muséum National d’Histoire Naturelle, 61 rue Buffon, 5 CP 53, 75231 Paris Cedex, France; 7 Newcastle University, School of Natural and Environmental Sciences, Newcastle‑upon‑Tyne, NE1 7RU UK; 8 UMR EIO (UPF-IRD-ILM-IFREMER), Université de La Polynésie Française, LabEx « Corail », BP 6570, 98702 Faa’a, Tahiti French Polynesia

Correction to: *Scientific Reports* 10.1038/s41598-021-00348-w, published online 25 October 2021

The Supplementary Information 1 and 2 files published with this Article contained errors, where the title was incorrectly given as “Multi-trophic marker analysis of a Marquesan food web highlights how reef ecosystems might respond to a warmer and nutrient-rich ocean future.”

This error has now been corrected in the Supplementary Information 1 and 2 files that accompany the original Article.

