# Supplementary material for: Multi-trophic markers illuminate the understanding of the functioning of a remote, low coral cover Marquesan coral reef food web
Source: Sci Rep. 2021 Oct 25;11:20950. doi: 10.1038/s41598-021-00348-w (PMC8545934; doi:10.1038/s41598-021-00348-w)
Supplement: Supplementary file 1 — Supplementary Information 1. [file 41598_2021_348_MOESM1_ESM.docx]

**Multi-trophic markers illuminate the understanding of the functioning of a remote, low coral cover Marquesan coral reef food web**

Pauline Fey^1^, Valeriano Parravicini^2^, Daniela Bănaru^3^, Jan Dierking^4^, René Galzin^2^, Benoit Lebreton^5^, Tarik Meziane^6^, Nicholas VC Polunin^7^, Mayalen Zubia^8^, Yves Letourneur^1*^

^1^ UMR ENTROPIE (UR-IRD-CNRS-IFREMER-UNC), LabEx « Corail », Université de la Nouvelle-Calédonie, BP R4, 98851 Nouméa Cedex, New Caledonia

^2^ CRIOBE, PSL Research University, USR 3278 EPHE-CNRS-UPVD, LabEx « Corail », Université de Perpignan, Avenue Paul Alduy, 66860 Perpignan Cedex, France (Orcid : 0000-0002-3408-1625)

^3^ Mediterranean Institute of Oceanography, UM 110 (AMU-UTV-CNRS-IRD), Campus de Luminy, Case 901, 13288 Marseille Cedex 9, France (Orcid : 0000-0002-8126-4328)

^4^ GEOMAR Helmholtz Centre for Ocean Research, Research Division Marine Ecology, Düsternbrooker Weg 20, 24105 Kiel, Germany (Orcid : 0000-0002-4660-6919)

^5^ UMR LIENSs 7266 (CNRS-ULR), Institut du littoral et de l’environnement, 2 rue Olympe de Gouges, 17000 La Rochelle, France (Orcid : 0000-0001-8802-2287)

^6^ Laboratoire BOREA, Muséum National d’Histoire Naturelle, CNRS 7208, IRD 207-SU-UCN-UA, Muséum National d’Histoire Naturelle, 61 rue Buffon, 5 CP 53, 75231 Paris Cedex, France (Orcid : 0000-0003-2142-6441)

^7^ Newcastle University, School of Natural and Environmental Sciences, Newcastle-upon-Tyne, NE1 7RU, United Kingdom

^8^ UMR EIO (UPF-IRD-ILM-IFREMER), Université de la Polynésie française, LabEx « Corail », BP 6570, 98702 Faa’a, Tahiti, French Polynesia (Orcid : [0000-0002-5043-3491](https://orcid.org/0000-0002-5043-3491))

^*^ Corresponding author: yves.letourneur@unc.nc ; Orcid : 0000-0003-3157-1976

**
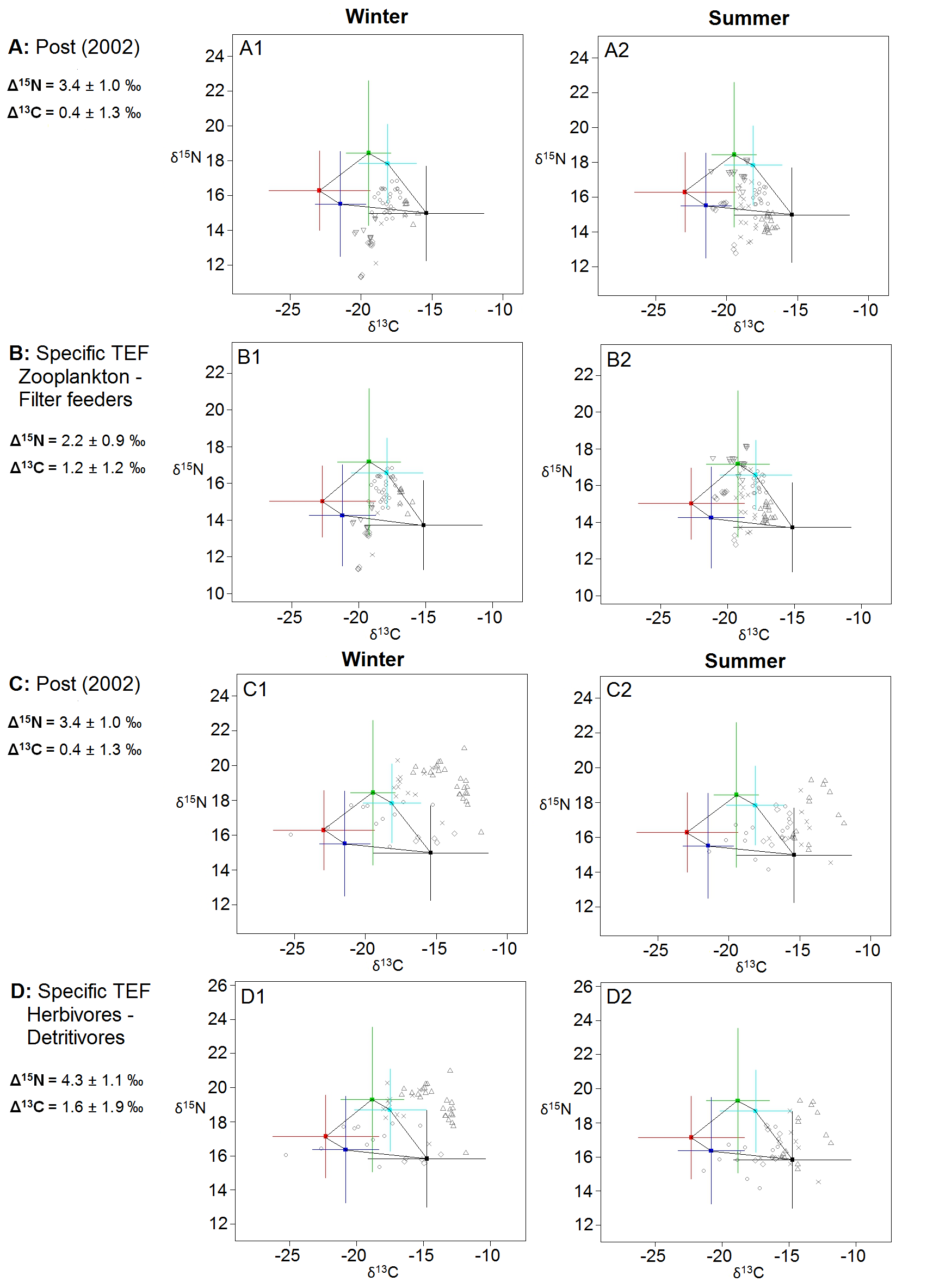
**

**Supplementary figure S1.** Comparison of the source polygons for the mixing models obtained with different trophic enrichment factors (TEF). For filter-feeders and zooplankton: fractionation factors of Post (2002) (A) and specific TEF (B). For herbivores and detritivores: fractionation factors of Post (2002) (C) and specific TEF (D). Cold season on the left, warm season on the right.


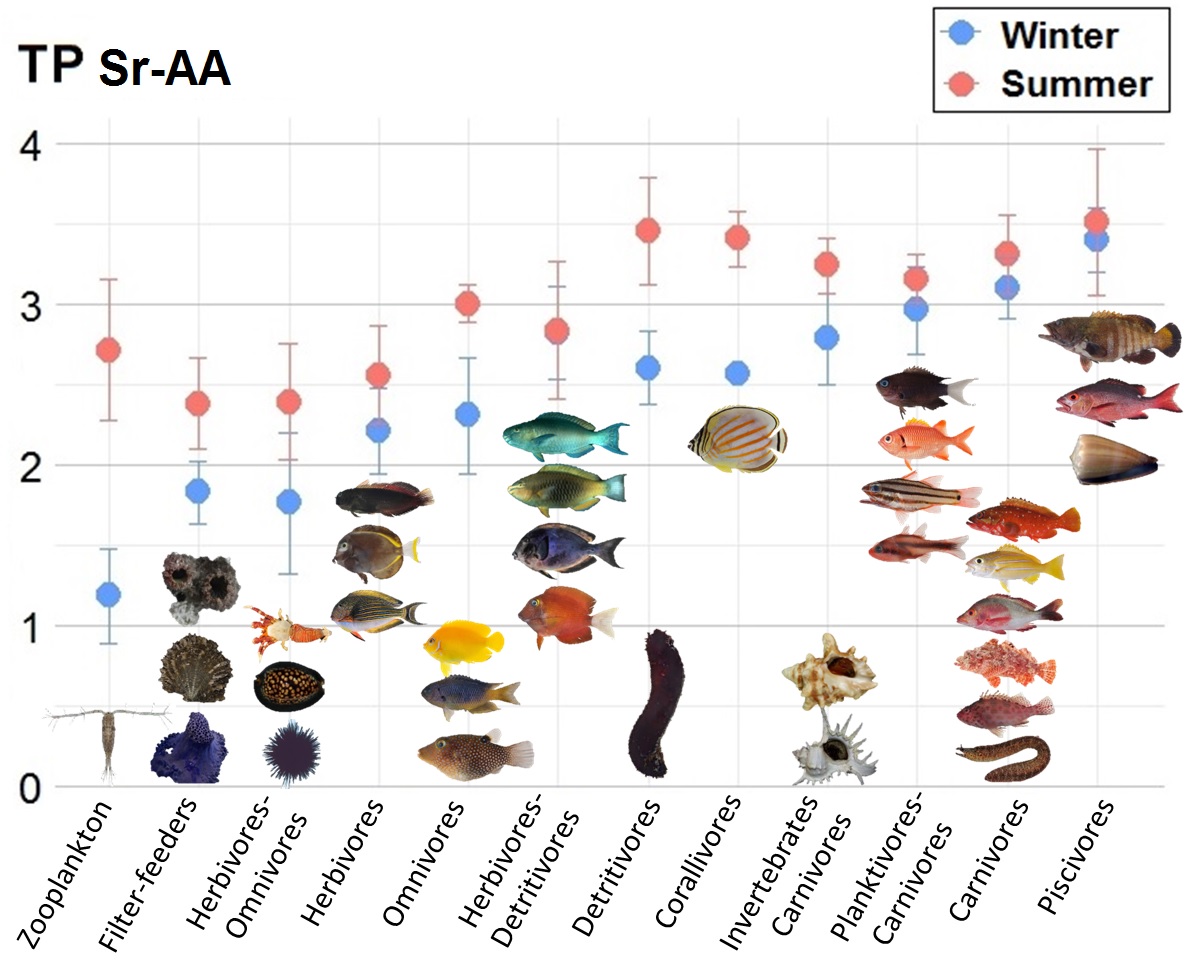


**Supplementary figure S2.** Trophic positions estimates for consumers by trophic group calculated with the Post equation (Post 2002) with the average δ^15^N of the source amino acids (glycine and phenylalanine) as baseline. Baseline value depending on the season: Winter δ^15^N_phe-gly_ = 12.8 ‰, Summer δ^15^N_phe-gly_ = 10.5 ‰.
