# Supplementary material for: Multi-trophic markers illuminate the understanding of the functioning of a remote, low coral cover Marquesan coral reef food web
Source: Sci Rep. 2021 Oct 25;11:20950. doi: 10.1038/s41598-021-00348-w (PMC8545934; doi:10.1038/s41598-021-00348-w)
Supplement: Supplementary file 2 — Supplementary Information 2. [file 41598_2021_348_MOESM2_ESM.docx]

**Multi-trophic markers illuminate the understanding of the functioning of a remote, low coral cover Marquesan coral reef food web**

Pauline Fey^1^, Valeriano Parravicini^2^, Daniela Bănaru^3^, Jan Dierking^4^, René Galzin^2^, Benoit Lebreton^5^, Tarik Meziane^6^, Nicholas VC Polunin^7^, Mayalen Zubia^8^, Yves Letourneur^1*^

^1^ UMR ENTROPIE (UR-IRD-CNRS-IFREMER-UNC), LabEx « Corail », Université de la Nouvelle-Calédonie, BP R4, 98851 Nouméa Cedex, New Caledonia

^2^ CRIOBE, PSL Research University, USR 3278 EPHE-CNRS-UPVD, LabEx « Corail », Université de Perpignan, Avenue Paul Alduy, 66860 Perpignan Cedex, France (Orcid : 0000-0002-3408-1625)

^3^ Mediterranean Institute of Oceanography, UM 110 (AMU-UTV-CNRS-IRD), Campus de Luminy, Case 901, 13288 Marseille Cedex 9, France (Orcid : 0000-0002-8126-4328)

^4^ GEOMAR Helmholtz Centre for Ocean Research, Research Division Marine Ecology, Düsternbrooker Weg 20, 24105 Kiel, Germany (Orcid : 0000-0002-4660-6919)

^5^ UMR LIENSs 7266 (CNRS-ULR), Institut du littoral et de l’environnement, 2 rue Olympe de Gouges, 17000 La Rochelle, France (Orcid : 0000-0001-8802-2287)

^6^ Laboratoire BOREA, Muséum National d’Histoire Naturelle, CNRS 7208, IRD 207-SU-UCN-UA, Muséum National d’Histoire Naturelle, 61 rue Buffon, 5 CP 53, 75231 Paris Cedex, France (Orcid : 0000-0003-2142-6441)

^7^ Newcastle University, School of Natural and Environmental Sciences, Newcastle-upon-Tyne, NE1 7RU, United Kingdom

^8^ UMR EIO (UPF-IRD-ILM-IFREMER), Université de la Polynésie française, LabEx « Corail », BP 6570, 98702 Faa’a, Tahiti, French Polynesia (Orcid : [0000-0002-5043-3491](https://orcid.org/0000-0002-5043-3491))

^*^ Corresponding author: yves.letourneur@unc.nc ; Orcid : 0000-0003-3157-1976

**Supplementary table S1.** Bulk isotopic compositions (δ^13^C and δ^15^N values) of organic matter (OM) sources, invertebrates and fish (mean ± SD). Data from cold and warm seasons are pooled. Trophic codes C: Carnivores, Cor: Corallivores, D: Detritivores, F: Filter-feeders, H: Herbivores, HD: Herbivores-Detritivores, HO: Herbivores-Omnivores, O: Omnivores, P: Piscivores, PkC: Planktivores-Carnivores, Z: Zooplankton.

|  |  |  | **Code** | **n** | **δ^13^C ‰** | **δ^15^N ‰** | **Trophic code** |
| --- | --- | --- | --- | --- | --- | --- | --- |
|  |  |  |  |  |  |  |  |
| **OM Pools** | Particulate organic matter | | POM | 38 | -22.5 ± 0.8 | 12.1 ± 1.1 |  |
|  | Sedimentary organic matter | | SOM | 30 | -19.1 ± 0.9 | 14.4 ± 0.5 |  |
| **Primary producers** | Detrital terrestrial plants | | DTP | 16 | -29.5 ± 1.7 | 1.9 ± 2.3 |  |
|  | Phytoplankton | | Phyto | 49 | -20.5 ± 0.6 | 15.0 ± 1.8 |  |
|  | Macroalgae | | Algae | 41 | -16.4 ± 1.9 | 11.6 ± 0.9 |  |
|  | Algal turf | | Turf | 32 | -23.9 ± 1.7 | 12.9 ± 0.6 |  |
|  |  | |  |  |  |  |  |
| **Invertebrates** | Ascidiidae | Ascidie sp. | Asc | 17 | - 18.6 ± 0.6 | 15.0 ± 1.0 | F |
|  | Clionaidae | *Spheciospongia* sp. | Spsp | 44 | - 17.9 ± 0.5 | 15.9 ± 0.6 | F |
|  | Pteriidae | *Pinctada margaritifera* | Pima | 24 | - 16.9 ± 0.3 | 14.7 ± 0.5 | F |
|  | Conidae | *Conus conco* | Coco | 9 | - 14.7 ± 0.9 | 20.4 ± 0.9 | P |
|  | Cypraeidae | *Mauritia* spp. | Maspp | 13 | - 16.0 ± 1.0 | 16.3 ± 0.8 | HO |
|  | Muricidae | *Chicoreus ramosus* | Chra | 16 | - 14.3 ± 0.6 | 18.2 ± 0.6 | C |
|  |  | *Mancinella armigera* | Maar | 13 | - 14.3 ± 1.1 | 18.7 ± 1.1 | C |
|  | Phyllidiidae | *Phyllidia cf madangensis* | Phspp | 5 | - 16.4 ± 1.1 | 13.6 ± 0.5 | HO |
|  |  | *Phyllidia varicosa* |  | 3 | - 15.1 ± 0.2 | 13.3 ± 0.6 |  |
|  | Diadematidae | *Echinothrix diadema* | Ecdi | 17 | - 14.8 ± 1.7 | 16.3 ± 1.5 | HO |
|  | Holothuridae | *Holothuria* sp. | Hosp | 6 | - 15.2 ± 0.6 | 18.5 ± 0.9 | D |
|  | Diogenidae | *Ciliopagurus vakovako* | Civak | 30 | - 17.8 ± 1.7 | 15.3 ± 0.8 | HO |
|  |  | *Dardanus sanguinocarpus* | Dasa | 17 | - 15.8 ± 1.9 | 14.4 ± 0.9 | HO |
|  | Zooplankton | 300-500 µm | Zoo-300 | 21 | - 19.9 ± 0.6 | 13.9 ± 1.6 | Z |
|  |  | 1000-2000 µm | Zoo-1000 | 28 | - 19.3 ± 0.6 | 15.9 ± 1.7 | Z |
|  |  | | | | | |  |
| **Fish** | Acanthuridae | *Acanthurus lineatus* | Acli | 14 | - 15.3 ± 1.5 | 15.7 ± 1.6 | H |
|  |  | *Acanthurus nigricans* | Acni | 25 | - 18.6 ± 2.4 | 16.5 ± 1.0 | H |
|  |  | *Acanthurus reversus* | Acre | 11 | - 17.2 ± 1.9 | 16.1 ± 0.7 | H |
|  |  | *Ctenochaetus flavicauda* | Ctfl | 11 | - 16.2 ± 1.3 | 17.4 ± 2.0 | HD |
|  |  | *Ctenochaetus marginatus* | Ctma | 38 | - 14.0 ± 1.2 | 18.5 ± 1.4 | HD |
|  | Apogonidae | *Apogon lativittatus* | Apla | 19 | - 17.0 ± 0.3 | 18.3 ± 1.3 | PkC |
|  |  | *Ostorhincus relativus* | Osre | 33 | - 17.0 ± 0.5 | 18.6 ± 1.2 | PkC |
|  | Blenniidae | *Cirripectes variolosus* | Civa | 20 | - 15.6 ± 1.4 | 16.2 ± 1.1 | H |
|  | Chaetodontidae | *Chaetodon ornatissimus* | Chor | 10 | - 12.5 ± 1.0 | 18.6 ± 0.6 | Cor |
|  |  | *Chaetodon trichrous* | Chtr | 15 | - 16.8 ± 1.0 | 18.7 ± 1.1 | PkC |
|  | Cirrhitidae | *Cirrhitichthys oxycephalus* | Ciox | 33 | - 16.8 ± 0.5 | 19.1 ± 1.0 | C |
|  | Holocentridae | *Myripristis berndti* | Mybe | 15 | - 16.7 ± 0.3 | 18.3 ± 0.8 | PkC |
|  |  | *Sargocentron tiere* | Sati | 22 | - 16.1 ± 0.4 | 19.4 ± 0.6 | C |
|  | Labridae | *Halichoeres claudia* | Hacl | 23 | - 17.0 ± 0.6 | 18.7 ± 1.3 | C |
|  | Lutjanidae | *Lutjanus bohar* | Lubo | 6 | - 15.9 ± 0.4 | 18.7 ± 2.7 | P |
|  |  | *Lutjanus gibbus* | Lugi | 10 | - 16.5 ± 0.9 | 19.3 ± 0.8 | C |
|  |  | *Lutjanus kasmira* | Luka | 20 | - 16.4 ± 0.6 | 19.0 ± 0.8 | C |
|  | Muraenidae | *Enchelycore pardalis* | Enpa | 4 | - 15.9 ± 0.5 | 19.4 ± 0.6 | C |
|  | Pomacanthidae | *Centropyge flavissima* | Cefl | 23 | - 18.4 ± 1.0 | 18.8 ± 0.7 | O |
|  | Pomacentridae | *Chromis abrupta* | Chab | 43 | - 17.4 ± 0.4 | 19.1 ± 0.9 | PkC |
|  |  | *Lepidozygus tapeinosoma* | Leta | 15 | - 17.4 ± 0.3 | 17.9 ± 0.9 | PkC |
|  |  | *Pomacentrus coelestis* | Poco | 19 | - 17.7 ± 0.4 | 17.0 ± 0.6 | O |
|  | Scarinae | *Scarus koputea* | Scar | 21 | - 16.1 ± 1.5 | 17.8 ± 1.6 | HD |
|  |  | *Scarus rubroviolaceus* |  |  |  |  | HD |
|  | Scorpaenidae | *Pterois antennata* | Ptan | 13 | -16.3 ± 0.5 | 18.7 ± 1.5 | C |
|  |  | *Scorpaenopsis possi* | Scpo | 18 | - 16.3 ± 0.4 | 18.7 ± 1.4 | C |
|  | Serranidae | *Cephalopholis argus* | Cear | 10 | - 15.3 ± 0.4 | 20.0 ± 0.4 | P |
|  |  | *Epinephelus fasciatus* | Epfa | 25 | - 16.3 ± 0.7 | 19.4 ± 1.1 | C |
|  | Tetraodontidae | *Canthigaster janthinoptera* | Caja | 13 | - 16.8 ± 0.5 | 17.6 ± 1.0 | O |
|  |  |  |  |  |  |  |  |

**Supplementary table S2.** Compositions in fatty acids trophic markers of primary consumers, expressed in % (± SD) of the sum of fatty acids in mg.g^-1^. Only fatty acids with an average proportion greater than 1 % for at least one sample group are presented.

| **Fatty acids (%)** | **Invertebrates** | | | | | **Fish** | | |
| --- | --- | --- | --- | --- | --- | --- | --- | --- |
|  | Zooplankton | Herbivore | Filter-feeders | | | Acanthuridae | | Scarinae |
|  |  | *Mauritia* spp. | *Pinctada margaritifera* | Ascidiidae | *Spheciospongia* sp. | *Acanthurus nigricans* | *Ctenochaetus marginatus* |  |
|  | n = 23 | n = 4 | n = 12 | n = 14 | n = 24 | n = 16 | n = 23 | n = 17 |
| **Saturated** |  |  |  |  |  |  |  |  |
| 12:0 | 0.34 ± 0.76 | 0.05 ± 0.02 | 0.11 ± 0.06 | 0.06 ± 0.06 | 1.38 ± 1.99 | 0.03 ± 0.02 | 0.06 ± 0.04 | 0.03 ± 0.02 |
| 14:0 | 12.07 ± 1.14 | 2.51 ± 0.25 | 1.25 ± 0.62 | 5.84 ± 2.18 | 1.96 ± 0.90 | 2.53 ± 0.88 | 4.36 ± 2.85 | 0.40 ± 0.11 |
| 15:0 | 1.09 ± 0.23 | 0.77 ± 0.13 | 15.88 ± 1.51 | 0.95 ± 0.17 | 1.88 ± 0.64 | 0.62 ± 0.09 | 0.53 ± 0.20 | 0.34 ± 0.06 |
| 16:0 | 21.67 ± 1.41 | 15.88 ± 1.51 | 17.54 ± 2.16 | 21.66 ± 3.84 | 11.67 ± 2.40 | 41.28 ± 5.00 | 23.87 ± 2.20 | 21.39 ± 1.27 |
| 17:0 | 1.22 ± 0.24 | 1.81 ± 0.20 | 1.42 ± 0.22 | 1.39 ± 0.22 | 3.08 ± 1.30 | 0.80 ± 0.15 | 0.69 ± 0.11 | 0.79 ± 0.09 |
| 18:0 | 5.89 ± 0.55 | 8.85 ± 0.84 | 8.15 ± 0.82 | 8.49 ± 1.81 | 7.12 ± 2.23 | 7.25 ± 1.40 | 9.46 ± 1.52 | 10.83 ± 0.65 |
| 20:0 | 0.43 ± 0.07 | 0.05 ± 0.04 | 0.05 ± 0.04 | 0.44 ± 0.13 | 1.93 ± 0.75 | 0.17 ± 0.04 | 0.18 ± 0.05 | 0.07 ± 0.03 |
| 21:0 | 0.06 ± 0.13 | - | 0.02 ± 0.07 | 0.03 ± 0.06 | 1.14 ± 2.73 | 0.01 ± 0.02 | 0.01 ± 0.02 | - |
| 22:0 | 0.31 ± 0.06 | 0.11 ± 0.21 | 0.09 ± 0.08 | 0.25 ± 0.08 | 2.45 ± 3.31 | 0.14 ± 0.05 | 0.19 ± 0.14 | 0.16 ± 0.31 |
| 24:0 | 0.20 ± 0.12 | 0.03 ± 0.04 | 0.03 ± 0.04 | 0.10 ± 0.03 | 1.64 ± 0.67 | 0.14 ± 0.05 | 0.22 ± 0.07 | 0.21 ± 0.12 |
| **Σ SFAs** | **43.58 ± 2.09** | **31.05 ± 1.06** | **29.02 ± 2.56** | **39.95 ± 3.54** | **35.97 ± 7.54** | **53.16 ± 4.36** | **40.02 ± 3.27** | **34.58 ± 1.10** |
|  |  |  |  |  |  |  |  |  |
| **Monounsaturated** | |  |  |  |  |  |  |  |
| 16:1ω7 | 8.48 ± 1.21 | 0.81 ± 0.11 | 1.49 ± 0.60 | 6.75 ± 1.65 | 1.58 ± 1.23 | 4.07 ± 1.71 | 6.05 ± 1.86 | 1.26 ± 0.23 |
| 16:1ω5 | 0.21 ± 0.04 | 2.73 ± 1.84 | 0.39 ± 0.86 | 0.25 ± 0.06 | 0.12 ± 0.16 | 0.20 ± 0.12 | 0.38 ± 0.07 | 0.19 ± 0.03 |
| 18:1ω9 | 3.96 ± 0.72 | 3.39 ± 0.38 | 10.72 ± 2.80 | 11.20 ± 2.60 | 3.99 ± 2.19 | 10.69 ± 1.43 | 6.57 ± 1.10 | 9.37 ± 1.18 |
| 18:1ω7 | 2.33 ± 0.27 | 0.91 ± 0.11 | 1.87 ± 0.29 | 2.92 ± 0.58 | 3.75 ± 1.40 | 1.98 ± 0.28 | 2.12 ± 0.46 | 2.53 ± 0.30 |
| 20:1ω11 | 0.22 ± 0.13 | 7.79 ± 0.94 | 10.29 ± 2.08 | 0.09 ± 0.04 | 0.39 ± 0.23 | 0.05 ± 0.03 | 0.09 ± 0.06 | 0.06 ± 0.01 |
| 20:1ω9 | 0.32 ± 0.10 | 0.44 ± 0.07 | 1.82 ± 0.35 | 0.17 ± 0.10 | 0.34 ± 0.27 | 1.96 ± 0.82 | 0.27 ± 0.13 | 0.13 ± 0.06 |
| 22:1ω11 | 0.07 ± 0.10 | 0.43 ± 0.15 | 0.20 ± 0.07 | 0.01 ± 0.02 | 3.23 ± 3.72 | 0.002 ± 0.006 | 0.005 ± 0.01 | 0.004 ± 0.02 |
| 22:1ω9 | 0.24 ± 0.16 | 0.46 ± 0.19 | 0.29 ± 0.13 | 0.05 ± 0.03 | 4.07 ± 3.77 | 0.17 ± 0.07 | 0.05 ± 0.04 | 0.03 ± 0.03 |
| **Σ MUFAs** | **16.44 ± 1.51** | **15.09 ± 1.61** | **27.32 ± 2.02** | **21.99 ± 1.19** | **18.04 ± 7.30** | **19.67 ± 3.90** | **15.98 ± 1.60** | **13.88 ± 1.06** |
|  |  |  |  |  |  |  |  |  |
| **Polyunsaturated** | |  |  |  |  |  |  |  |
| 16:2ω4 | 0.87 ± 0.22 | 0.02 ± 0.02 | 0.06 ± 0.04 | 0.31 ± 0.11 | 0.69 ± 0.90 | 0.05 ± 0.03 | 0.45 ± 0.25 | 0.04 ± 0.02 |
| 16:3ω4 | 0.66 ± 0.36 | 0.02 ± 0.03 | 0.10 ± 0.13 | 0.07 ± 0.05 | 0.12 ± 0.22 | 0.06 ± 0.03 | 1.45 ± 0.84 | 0.04 ± 0.02 |
| 18:2ω6 | 1.95 ± 0.47 | 3.38 ± 0.17 | 3.16 ± 0.73 | 2.59 ± 0.19 | 1.35 ± 0.52 | 0.89 ± 0.25 | 1.54 ± 0.16 | 2.23 ± 0.48 |
| 18:3ω3 | 0.73 ± 0.27 | 1.71 ± 0.23 | 0.58 ± 0.12 | 0.63 ± 0.13 | 3.68 ± 2.49 | 0.33 ± 0.12 | 0.93 ± 0.35 | 0.45 ± 0.25 |
| 18:4ω3 | 1.02 ± 0.16 | 0.33 ± 0.14 | 0.57 ± 0.19 | 1.20 ± 0.49 | 0.20 ± 0.20 | 0.14 ± 0.07 | 1.72 ± 0.81 | 0.24 ± 0.12 |
| 20:2ω6 | 0.23 ± 0.05 | 1.07 ± 0.15 | 0.32 ± 0.07 | 0.27 ± 0.05 | 1.97 ± 0.53 | 0.14 ± 0.04 | 0.21 ± 0.05 | 0.39 ± 0.10 |
| 20:3ω6 | 0.32 ± 0.08 | 0.11 ± 0.08 | 0.19 ± 0.06 | 0.41 ± 0.08 | 1.87 ± 1.00 | 1.04 ± 0.28 | 0.59 ± 0.11 | 0.93 ± 0.14 |
| 20:4ω6 | 2.98 ± 0.33 | 13.24 ± 0.98 | 7.95 ± 1.28 | 7.21 ± 3.44 | 24.47 ± 8.12 | 7.13 ± 3.04 | 11.14 ± 3.70 | 22.28 ± 2.20 |
| 20:4ω3 | 0.35 ± 0.06 | 0.02 ± 0.03 | 0.08 ± 0.03 | 0.30 ± 0.07 | 1.07 ± 0.48 | 0.25 ± 0.07 | 0.24 ± 0.07 | 0.20 ± 0.09 |
| 20:5ω3 | 12.03 ± 1.89 | 1.97 ± 0.20 | 2.89 ± 0.66 | 10.89 ± 1.26 | 2.87 ± 1.26 | 6.31 ± 1.68 | 6.04 ± 1.11 | 7.23 ± 1.20 |
| 22:2ω9 | 0.004 ± 0.01 | 6.80 ± 4.56 | 4.93 ± 2.78 | 0.01 ± 0.01 | 0.19 ± 0.21 | 0.02 ± 0.05 | 0.06 ± 0.11 | 0.05 ± 0.06 |
| 22:2ω6 | 0.01 ± 0.03 | 1.26 ± 0.88 | 1.11 ± 0.63 | 0.01 ± 0.01 | 0.29 ± 0.76 | 0.05 ± 0.11 | 0.02 ± 0.03 | 0.01 ± 0.01 |
| 22:4ω6 | 0.17 ± 0.08 | 9.39 ± 0.75 | 1.76 ± 0.48 | 0.30 ± 0.10 | 0.34 ± 0.58 | 1.90 ± 0.37 | 1.35 ± 0.16 | 1.46 ± 0.19 |
| 22:5ω6 | 0.96 ± 0.18 | 0.92 ± 0.38 | 3.07 ± 0.62 | 1.68 ± 0.63 | 0.23 ± 0.30 | 0.77 ± 0.33 | 2.37 ± 0.72 | 3.07 ± 0.72 |
| 22:5ω3 | 0.77 ± 0.31 | 9.07 ± 1.30 | 0.99 ± 0.27 | 0.41 ± 0.07 | 0.17 ± 0.29 | 3.03 ± 0.92 | 2.63 ± 0.62 | 2.40 ± 0.31 |
| 22:6ω3 | 14.66 ± 2.41 | 0.97 ± 0.43 | 13.67 ± 1.15 | 8.05 ± 1.29 | 0.83 ± 0.79 | 4.37 ± 1.87 | 11.03 ± 2.99 | 9.60 ± 1.70 |
| **Σ PUFAs** | **38.65 ± 2.43** | **52.15 ± 2.45** | **42.72 ± 2.36** | **35.50 ± 3.48** | **43.06 ± 7.20** | **26.93 ± 7.94** | **43.24 ± 4.82** | **51.20 ± 1.76** |
|  |  |  |  |  |  |  |  |  |
| **Branched** |  |  |  |  |  |  |  |  |
| 17:0iso | 0.34 ± 0.04 | 0.34 ± 0.09 | 0.16 ± 0.07 | 0.67 ± 0.22 | 1.55 ± 0.75 | 0.07 ± 0.02 | 0.12 ± 0.03 | 0.12 ± 0.03 |
| **Σ BrFAs** | **1.17 ± 0.13** | **1.17 ± 0.19** | **0.56 ± 0.28** | **2.46 ± 0.49** | **2.89 ± 0.81** | **0.24 ± 0.06** | **0.64 ± 0.28** | **0.33 ± 0.10** |
|  |  |  |  |  |  |  |  |  |
| **Σ FA** (mg.g^-1^) | **26.13 ± 16.42** | **3.73 ± 1.85** | **2.68 ± 0.74** | **17.29 ± 7.54** | **0.31 ± 0.10** | **26.03 ± 18.82** | **8.93 ± 3.55** | **6.80 ± 2.14** |

**Supplementary table S3.** Bulk isotopic compositions (δ^13^C and δ^15^N values) of secondary consumers used for amino acids analyses (mean ± SD). Cold season in white, warm season in light grey.

|  |  | Code | n | δ^13^C ‰ | δ^15^N ‰ |
| --- | --- | --- | --- | --- | --- |
|  |  |  |  |  |  |
| **Invertebrate** |  |  |  |  |  |
| Conidae | *Conus conco* | Coco | 6 | - 15.2 ± 0.5 | 20.9 ± 0.7 |
|  |  |  | 3 | - 13.7 ± 0.6 | 19.4 ± 0.4 |
| **Fish** | |  |  |  |  |
| Lutjanidae | *Lutjanus bohar* | Lubo | 2 | - 15.6 ± 0.6 | 21.9 ± 0.5 |
|  |  |  | 4 | - 16.0 ± 0.1 | 17.1 ± 1.4 |
|  | *Lutjanus gibbus* | Lugi | 4 | - 15.5 ± 0.4 | 20.1 ± 0.5 |
|  |  |  | 6 | - 17.1 ± 0.6 | 18.7 ± 0.4 |
|  | *Lutjanus kasmira* | Luka | 3 | - 15.9 ± 0.1 | 20.3 ± 0.7 |
|  |  |  | 17 | - 16.5 ± 0.6 | 18.8 ± 0.6 |
| Muraenidae | *Enchelycore pardalis* | Enpa | 2 | - 15.6 ± 0.2 | 20.0 ± 0.2 |
|  |  |  | 2 | - 16.2 ± 0.6 | 18.9 ± 0.2 |
| Scorpaenidae | *Scorpaenopsis possi* | Scpo | 10 | - 16.0 ± 0.4 | 19.8 ± 0.9 |
|  |  |  | 8 | - 16.6 ± 0.3 | 17.3 ± 0.3 |
| Serranidae | *Cephalopholis argus* | Cear | 4 | - 15.2 ± 0.3 | 20.6 ± 0.1 |
|  |  |  | 6 | - 15.3 ± 0.5 | 20.1 ± 0.3 |
|  | *Epinephelus fasciatus* | Epfa | 10 | - 15.8 ± 0.3 | 20.3 ± 0.5 |
|  |  |  | 15 | - 16.6 ± 0.7 | 18.7 ± 0.9 |
